# New *Trypanosoma brucei* acting derivatives incorporating 1-(4-phenyl)adamantane and 1-(4-phenoxyphenyl)adamantane†

**DOI:** 10.1039/d5md00135h

**Published:** 2025-04-25

**Authors:** Konstantina Stavropoulou, Angeliki Kaimaki, Maria Nikolaou, Ana K. Brown, Andrew Tsotinis, Martin C. Taylor, John M. Kelly, Ioannis P. Papanastasiou

**Affiliations:** a Division of Pharmaceutical Chemistry, Department of Pharmacy, School of Health Sciences, National and Kapodistrian University of Athens Panepistimiopoli-Zografou 157 71 Athens Greece papanastasiou@pharm.uoa.gr; b Department of Chemistry, University of San Francisco 2130 Fulton Street San Francisco California CA 94117 USA; c Department of Pathogen Molecular Biology, London School of Hygiene and Tropical Medicine Keppel Street London WC1 E7HT UK

## Abstract

In this work, we describe the design, synthesis and evaluation of novel functionalised 1-(4-phenyl)adamantane and 1-(4-phenoxyphenyl)adamantane derivatives. Based on previous findings, we incorporated a phenyl ring between the adamantane core and the pharmacophoric side chain to enhance the activity and selectivity index (SI). The aromatic imidazolines 1a–d and the linear amidines 2a,b and 3a,b exhibited notable activity against *T. brucei*. The 1-(4-phenyl)adamantane 1-(4-phenoxyphenyl)adamantane core was further functionalized with the aminoguanylhydrazone and thiosemicarbazone moieties. 2-[(*E*)-4-(1-adamantyl)benzylidene]hydrazine-1-carbothioamide 4c emerged as a promising trypanocidal agent with an EC_50_ of 0.16 μM and an SI of 17. Future studies will focus on optimizing the length and the distance of the side chain between the aromatic ring and the chromophores to further enhance the activity and selectivity of these molecules.

## Abstract 2

New 1-(4-phenyl)adamantane and 1-(4-phenoxyphenyl)adamantane derivatives, functionalised with imidazolines, linear amidines, aminoguanylhydrazone and thiosemicarbazone moieties present activity against *T. brucei*.

## Graphical abstract

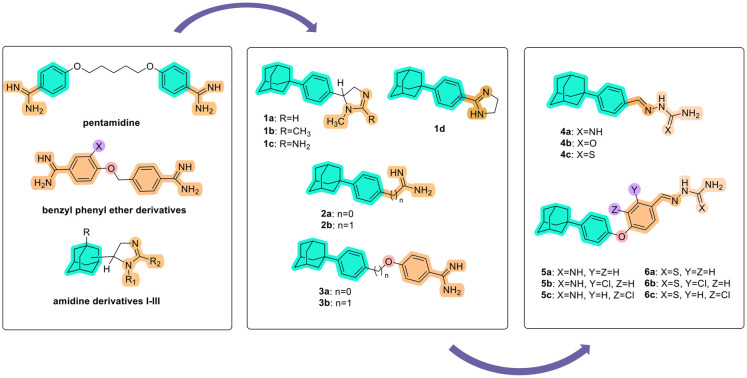

## Introduction

Human African Trypanosomiasis (HAT) is a neglected tropical disease endemic to sub-Saharan Africa. Although detected cases have recently dropped below 1000 annually,^1^ the risk of large epidemic outbreaks remains high, and trypanosome infections continue to impose a large disease burden on domestic livestock throughout the region. The treatment of late-stage HAT (when parasites have accessed the CNS) with nifurtimox–eflornithine combination therapy (NECT) requires systematic hospitalization, which poses challenges in resource-limited areas. The recent introduction of fexinidazole as an oral drug has therefore been an important advance.^2^ However, further chemotherapeutic options are needed to maintain progress towards elimination of the disease as a public health problem, and there remains a demand for new veterinary drugs.

Pentamidine is a diamidine that has been used in the treatment of stage 1 (hemolymphatic) gambiense HAT for nearly 80 years. Several mechanisms have been suggested for pentamidine activity, but the precise mode of action and its main targets have not been entirely elucidated. Pentamidine is believed to be a DNA minor groove binder, particularly to AT-rich regions. However, antimicrobial activity of pentamidine does not correlate well with the DNA binding action.^3,4^ In addition, pentamidine interrupts tRNA aminoacylation, by the entropy-driven non-specific binding, which inhibits translation.^5^ The drug has also been shown to inhibit enzymes such as topoisomerases of both *Pneumocystis carinii* and African trypanosomes,^6,7^ the phosphatase of regenerating liver,^8^ and to selectively modify ubiquitin.^9^ Resistance to pentamidine is associated with an inability of the diamidine to reach its target(s). The aquaglyceroporin TbAQP2 has been demonstrated to be the major mediator of pentamidine uptake,^10–12^ and to correspond to the previously identified high-affinity pentamidine transporter (HAPT 1). Other transporters also contribute to a lesser extent, including TbAT1, LAPT1 (ref. 13) and P-glycoprotein (P-gp)-like and organic cation transporters (OCT).^14^ The trypanosome P2 transporter (TbAT1) is substantially different from the analogous human mammalian nucleoside transporters.^15^ However, the P2 transporter has a recognition motif, which is common between the amino-purines, the melamine-based arsenicals and the diamidines, and consists of an amidine moiety, an aromatic ring and an electronegative heteroatom.^16^

**Fig. 1:**
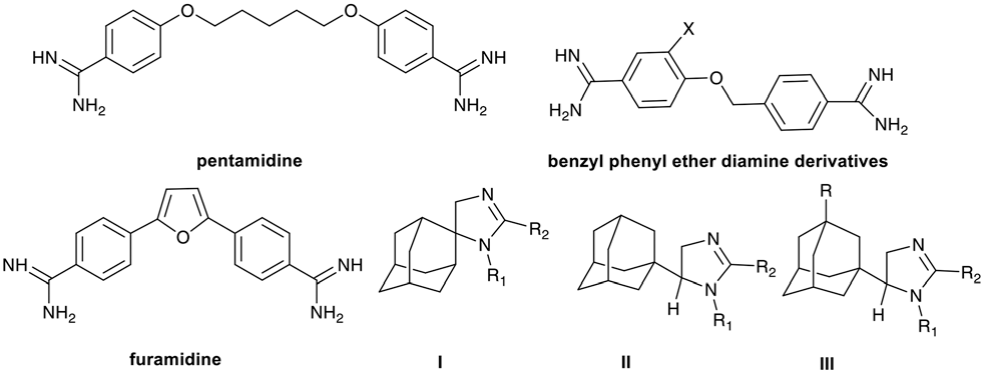
Pentamidine, furamidine and other amidine derivatives.

In this study, we present the synthesis of new aromatic amidines, building on our previous findings and exploring the chemical space of adamantane derivatives and their biological potential against trypanosomes. In our earlier work, we synthesized spiro **I** and non-spiro **II** and **III** imidazoline derivatives, each incorporating an amidine moiety within the scaffold, along with the lipophilic adamantane core (Fig. 1).^17,18^ We have also observed that inserting a phenyl ring between the adamantane core and the functional side chain improved, in certain cases, the activity and selectivity.^19^ Bis(arylimidamides) are known to target kinetoplastid parasites.^20^ In the present work, we modified the imidazoline scaffold to include the aromatic imidazolines 1a–d and the exocyclic aromatic amidines 2a,b. Additionally, we introduced a benzylphenylether spacer, previously employed in related studies,^21^ into the 3a amidine.

**Fig. 2:**
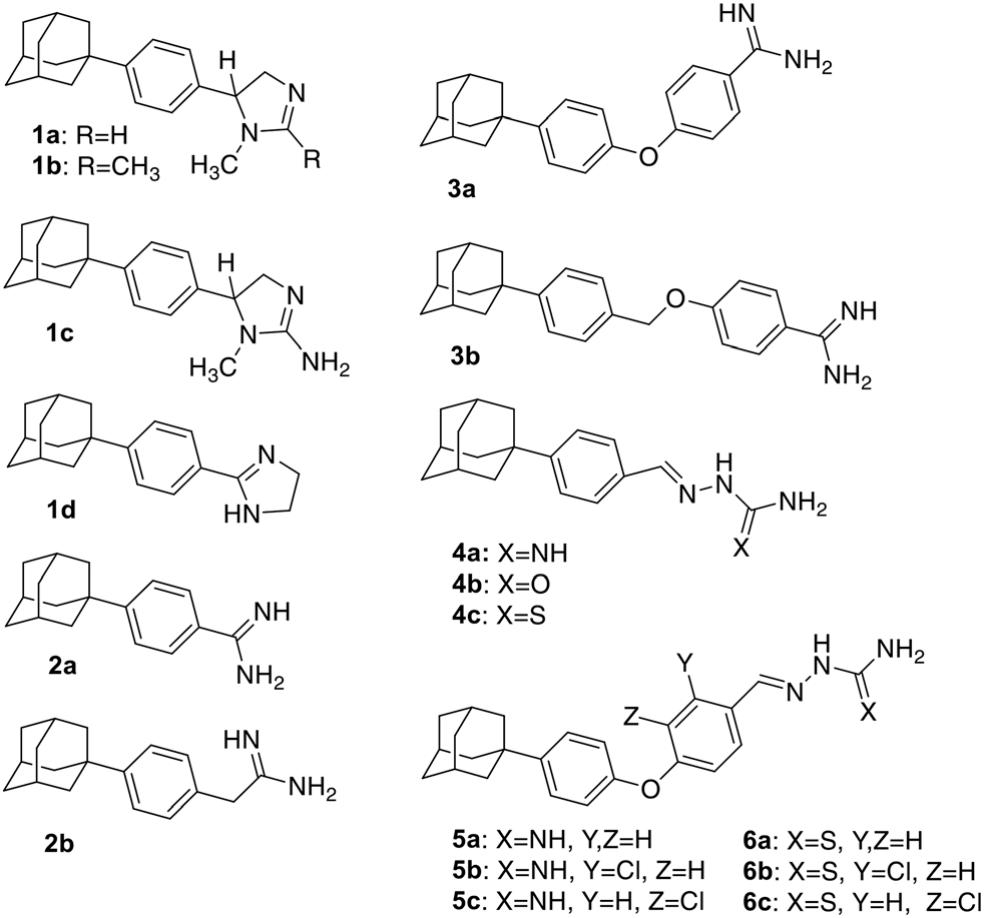
New 1-(4-phenyl)adamantane, 1-(4-phenoxyphenyl) adamantane and 1-(4-benzyloxyphenyl)adamantane functionalized derivatives with trypanocidal activity.

Imines and their congeneric classes of compounds, such as Schiff bases, thio/semicarbazones, hydrazones, and benzopyrazines, are endowed with biological activity, which has been exploited in drug development. Therefore, the side chains of the new derivatives were further functionalized with aminoguanylhydrazone and thiosemicarbazone groups, affording the derivatives 4a–c, 5a–c and 6a–c. These two pharmacophores are well-known for their trypanocidal activity,^22–25^ among other properties.^26–29^ In particular, aromatic adamantane secondary thiosemicarbazones exhibit antimicrobial activity.^30^ Moreover, the incorporation of a halogen atom onto the aromatic ring may enhance biological activity or reduce toxicity, as observed in similar aminoguanylhydrazones.^31^ This approach was applied to derivatives 5a–c and 6a–c (Fig. 2).

## Results and discussion

### Chemistry

**Scheme 1:**
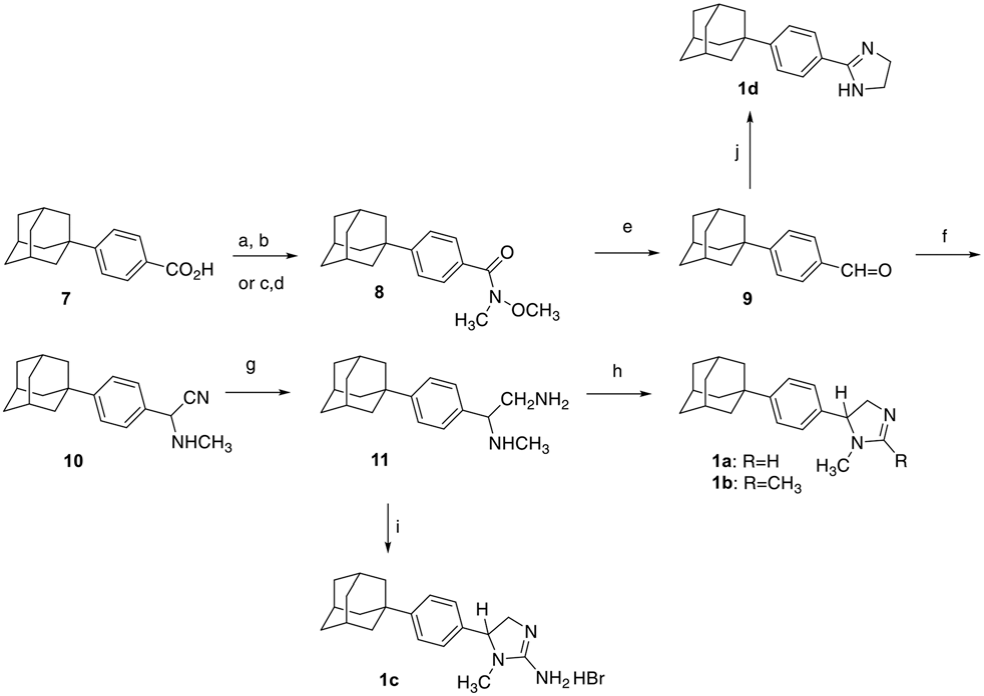
Reagents and conditions: (a) N(CH_2_CH_2_OMe)_2_SF_3_, DIPEA, DCM, 0 °C, 30 min; (b) HN(OMe)Me·HCl, DIPEA, DCM, 0 °C, 30 min, rt for 24 h, 55 °C for 24 h; c) PPh_3_, NBS, 0 °C, 15 min; (d) HN(OMe)Me·HCl, EtN_3_, DCM, rt, 3 h; (e) LDBBA, THF, 0 °C, 30 min; (f) NaCN, CH_3_NH_2_·HCl, DMSO : H_2_O, 9 : 1, 60 °C, 7 h; (g) H_2_/PtO_2_, EtOH, HCl(g)/EtOH, 55 psi, rt, 4.5 h; (h) formamidine acetate or acetamidine hydrochloride, EtOH, reflux, 48 h; (i) BrCN, DCM, rt, 48 h; (j) I_2_, ethylenediamine, K_2_CO_3_, *tert*-BuOH, 70 °C, 3 h.

The preparation of the 4-phenyl-2-imidazolines 1a–d was realized by the reaction sequence illustrated in Scheme 1.

For the preparation of the 4-phenyl-2-imidazolines 1a–d, the key compound 4-(1-adamantyl)benzaldehyde (9)^32^ was converted to the *a*-aminoacetonitrile 10*via* the Strecker reaction.^33,34^ The latter was hydrogenated to the corresponding diamine 11, which upon treatment with the requisite formamidine acetate, acetamidine hydrochloride or cyanogen bromide afforded the imidazolines 1a–c, respectively.^17^ The benzaldehyde 9 was converted to the 2-imidazoline 1d upon treatment with iodine, ethylenediamine and potassium carbonate.^35^ In this work, we also report the smooth reduction of the Weinreb amide 8 by lithium diisobutyl-*tert*-butoxyaluminum hydride (LDBBA)^36^ to the respective benzaldehyde 9 and two different ways for the Weinreb amide 8^37^ preparation. One route, involved the activation of the benzoic acid 7^38^ by the Deoxo-Fluor reagent and then treatment with *N*,*O*-dimethylhydroxylamine.^39,40^ In the second preparation, the benzoic acid 7 was transformed to the intermediate alkyloxyphosphonium salt, *via* an Appel type reaction with NBS and TPP, which was then treated with *N*,*O*-dimethylhydroxylamine.^41^

**Scheme 2:**
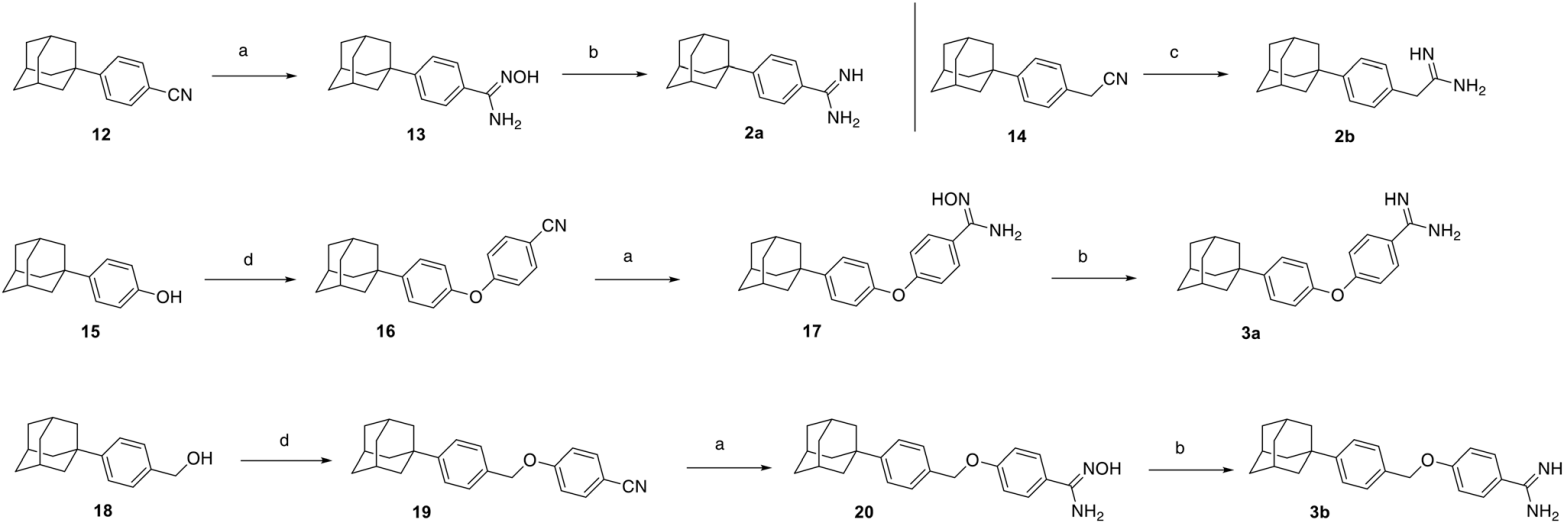
Reagents and conditions: (a) i. NH_2_OH·HCl, DIPEA, EtOH, rt, 1 h; ii. EtOH, reflux, 16 h; (b) i. Ac_2_O, AcOH, rt, 1.5 h; ii. Zn, rt, 24 h; iii. NaOH 5 M; (c) i. NH_4_Cl, Al(Me)_3_, toluene dry, 2 h, 0 °C to rt; ii. 80 °C, 19 h; iii. SiO_2_, DCM, rt, 10 min; (d) i. NaH, DMF dry, rt, 10 min; ii. 4-fluorobenzonitrile, DMF dry, 100 °C, 24 h.

The linear aromatic adamantane amidines 2a,b and 3a,b were prepared as shown in Scheme 2. We adopted a facile and efficacious method for the synthesis of amidines 2a and 3a,b from the corresponding nitriles 12,^42^14, 16 and 19*via* the reduction of the intermediate amidoximes, in the presence of Ac_2_O/AcOH/Zn at room temperature.^43^ Acetamidine 2b was afforded directly from the phenoacetonitrile 14 (ref. 32) upon treatment with the aluminum amide, reagent generated *in situ*.^44^ The 4-phenoxyphenylnitrile 15 and the 4-benzyloxyphenylnitrile 19 were afforded by a nucleophilic aromatic substitution of the requisite alcohols 15 (ref. 32) and 18 (ref. 32) to the 4-fluorobenzonitrile.

**Scheme 3:**
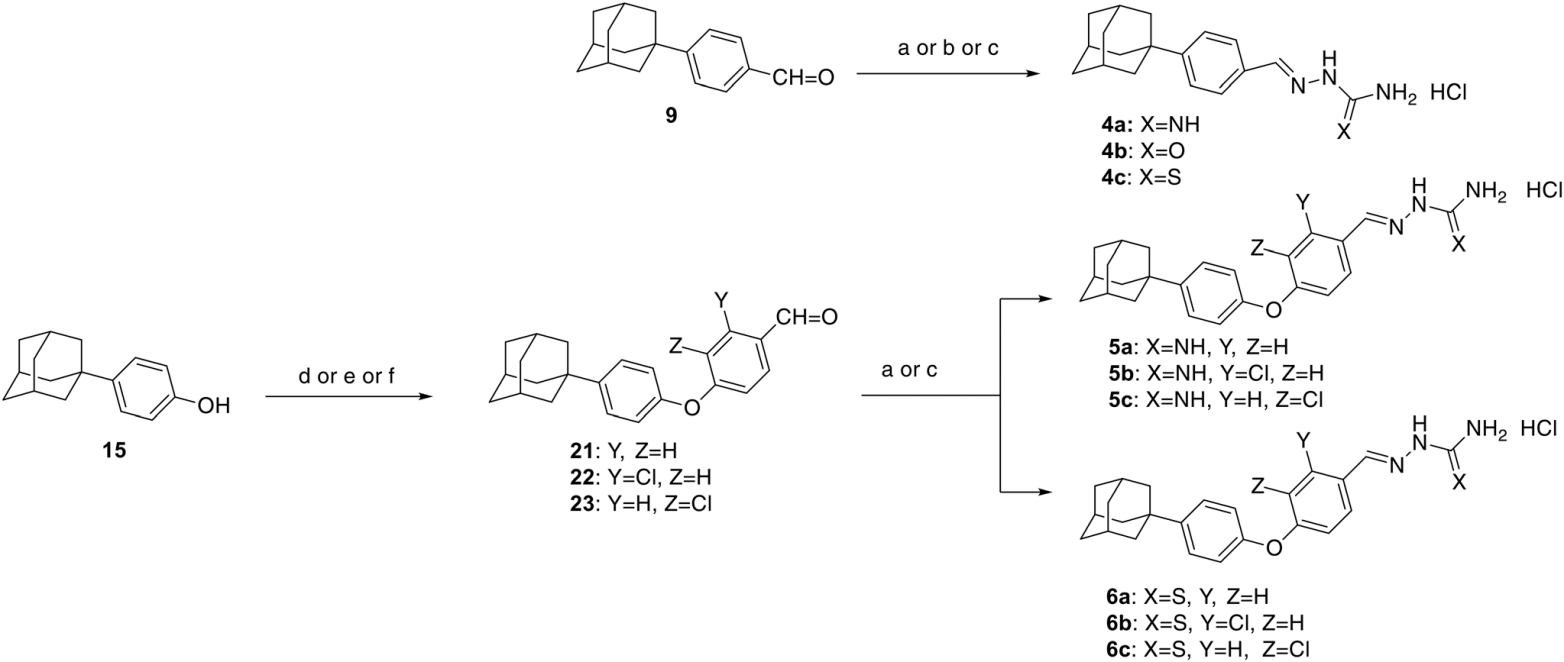
Reagents and conditions: (a) aminoguanidine bicarbonate, EtOH, conc. HCl drops, 18 h; (b) semicarbazide hydrochloride, EtOH, HCl drops, 18 h; (c) thiosemicarbazide, EtOH, conc. HCl drops, 18 h; (d) K_2_CO_3_, DMF dry, 2-chloro-4-fluorobenzaldeyde, 160 °C, 24 h; (e) K_2_CO_3_, DMF dry, 4-fluorobenzaldeyde, 160 °C, 24 h; (f) K_2_CO_3_, DMF dry, 3-chloro-4-fluorobenzaldeyde, 160 °C, 24 h.

The synthesis of the aminoguanylhydrazones and thiosemicarbazones 4a–c, 5a–c and 6a–c is depicted in Scheme 3. The parent benzaldehydes 9, 21–23 were coupled with aminoguanidine bicarbonate, semicarbazide hydrochloride and thiosemicarbazide, respectively, leading to the corresponding final derivatives 4a–c, 5a–c and 6a–c. The 4-phenoxybenzaldehydes 21–23 were prepared from the phenol 15 in a similar way^26^ to the aforementioned 4-phenoxyphenylnitrile 16.

### Pharmacology

The new adamantane adducts were tested for their activity against the bloodstream-form *Trypanosoma brucei* and the results are listed in Table 1.

**Table 1:** Activity and cytotoxicity of the new derivatives

Cmpd	*T. brucei* EC_50_^a^ (μM)	*T. brucei* EC_90_^a^ (μM)	HeLa cells EC_50_ (μM)	SI^b^
1a	0.93 ± 0.05	1.42 ± 0.15	3.68 ± 0.12^c^	4.3
1b	1.39 ± 0.03	1.69 ± 0.02	5.59 ± 0.94^c^	4.0
1c	1.58 ± 0.05	2.11 ± 0.16	7.58 ± 0.64^c^	4.8
1d	1.54 ± 0.05	2.07 ± 0.08	3.06 ± 0.34^c^	2.0
2a	1.15 ± 0.19	1.90 ± 0.37	1.55 ± 0.13	1.3
2b	0.67 ± 0.02	0.86 ± 0.02	2.04 ± 0.18	3.0
3a	0.27 ± 0.01	0.34 ± 0.01	0.82 ± 0.10	3.0
3b	0.69 ± 0.02	0.99 ± 0.20	0.93 ± 0.06	1.3
4a	0.36 ± 0.03	0.44 ± 0.03	1.17 ± 0.09	3.3
4b	>100	>100	>100	—
4c	0.16 ± 0.02	0.27 ± 0.01	2.77 ± 0.14	17
5a	0.34 ± 0.01	0.45 ± 0.01	0.54 ± 0.01	1.6
5b	0.70 ± 0.10	1.02 ± 0.02	<0.22	<1
5c	0.33 ± 0.03	0.51 ± 0.01	<0.22	<1
6a	>35	>35	—	—
6b	0.45 ± 0.03	0.88 ± 0.04	0.26 ± 0.04	<1
6c	1.39 ± 0.09	2.44 ± 0.32	2.08 ± 0.21	1.5
**Pent**	0.0019 ± 0.0001	0.0044 ± 0.0001	5.70 ± 0.61	3000

a EC_50_/EC_90_; concentrations that inhibit growth by 50% and 90%.

b S.I.; selectivity index, the ratio of EC_50_ values obtained with HeLa cells and *T. brucei*.

c Cytotoxicity was established using L6 cells in these cases; Pent, pentamidine.

It is apparent from the test results that the 2-imidazolines 1a–d are less cytotoxic than their linear congeners 2a,b and 3a,b, although the latter are generally more potent. The insertion of the phenyl ring between the adamantane core and the side chain confirmed the improvement of the antiparasitic potency of the 5-phenyl-2-imidazolines *versus* their congeners, with a phenyl substitution at C3 of adamantane or *N*1 of imidazolines.^34^ Additionally, the linker connecting the two aromatic rings appears to affect the pharmacological profile of these compounds. A two-atom distance (C, O) between the two aromatic rings lowers the activity as is evident by comparing the 4-benzyloxyphenyl amidine 3b and the 4-phenyloxyphenyl amidine 3a. A bioisosteric replacement of the oxygen atom between the two rings is a promising avenue for future development of these derivatives.

The relative distance between the phenyl ring and the functional group at the side chain has an impact on both the activity and cytotoxicity. Notably, the acetamidine 2b is more potent than benzamidine 2a and the diaryl amidine 3a presents even higher activity. These results prompted us to further explore the impact of the length and the distance of the side chain between the aromatic ring and the functional group. Amongst the aminoguanylhydrazones and thiosemicarbazones 4a–c, 5a–c and 6a–c, the monoaryl adducts were better tolerated. The thiosemicarbazone 4c exhibited the most promising pharmacological profile among these series of derivatives with a higher SI. This derivative has been also identified as antimycobacterial in a whole cell high-throughput screen.^45^ In contrast, the semicarbazone moiety (adduct 4b) has no trypanocidal effect. The impact of chlorine substitution on activity remains unclear in these derivatives, even though the non-substituted thiosemicarbazone 6a is less potent than its chlorinated congeners 6b and c. Additionally, the aromatic adamantane aminoguanylhydrazones and thiosemicarbazones reported here are more potent than the previously described non-aromatic adamantane.^46,47^

## Conclusions

The results of the current study confirm that the insertion of a phenyl ring between the adamantane core and the side chain improves the pharmacological profile of 5-phenyl-2-imidazolines compared to those with phenyl substitutions at C3 or *N*1. The linker between the two aromatic rings also affects activity, with a two-atom (C, O) distance reducing potency and increasing cytotoxicity. Future studies could explore bioisosteric modifications replacing the oxygen atom. Among the aminoguanylhydrazone and thiosemicarbazone functionalised derivatives, thiosemicarbazone 4c has shown the most promising pharmacological profile. Additionally, the distance between the phenyl ring and the functional group in the side chain affects both activity and cytotoxicity. These results suggest that further investigation into optimizing the side chain length and the relative position of the structural features is warranted.

### Biology

#### Cytotoxic activity against mammalian cells

Cytotoxicity against mammalian cells (HeLa or L6, a rat skeletal myoblast line) was assessed using microtitre plates.§§HeLa and L6 cells were obtained from the London School of Hygiene and Tropical Medicine (LSHTM) cell line repository. Briefly, cells were seeded at 1 × 10^4^ mL^−1^ in 200 μL of growth medium containing 7 different compound concentrations in a range previously established to encompass both the EC_50_ and EC_90_ values. The plates were incubated for 6 days at 37 °C and 20 μL resazurin (at 0.125 mg mL^−1^) was then added to each well. After an additional 8 hours incubation, the fluorescence was determined using a FLUOstar Omega fluorescent plate reader (BMG Labtech). Inhibition of growth was calculated by comparison with control values and EC_50_ and EC_90_ values were determined in triplicate using linear regression analysis.

#### *Trypanosoma brucei* culturing and drug testing

Bloodstream form *T. brucei* (strain 427) were cultured at 37 °C in modified Iscove's medium. Trypanocidal activity was assessed by growing parasites in microtiter plates in the presence of various drug concentrations. Parasites were seeded at 0.25 × 10^5^ mL^−1^ in 200 μL of growth medium containing 7 different compound concentrations in a range previously established to encompass both the EC_50_ and EC_90_ values. The plates were incubated for 48 hours at 37 °C and 20 μL resazurin (as above) was then added to each well. After an additional overnight incubation, the fluorescence was determined using a FLUOstar Omega fluorescent plate reader, and growth inhibition calculated. Values were determined in triplicate using linear regression analysis.

#### Synthetic procedures

All chemicals and solvents were obtained from commercial suppliers and used without further purification. Reactions were monitored by thin layer chromatography. Melting points were determined on a Sanyo Gallenkamp apparatus and are uncorrected. Infrared (IR) spectra were recorded on a Perkin-Elmer 833 spectrophotometer. ^1^H-NMR spectra recorded on a Bruker DRX 400 (400 MHz) spectrometer and ^13^C-NMR spectra were taken at 50 MHz on Bruker AC 200 (200 MHz) spectrometer and at 150 MHz on Bruker Avance 600 spectrometer (600 MHz). All NMR spectra were taken in deuterochloroform or hexadeuterodimethyl sulfoxide and the chemical shifts are reported in ppm. Elemental analyses (C, H, N) were carried out by the Institute of Chemical Biology, NHRF, Greece and the results obtained had a maximum deviation of ±0.4% from the theoretical value.

##### 4-(1-Tricyclo[3.3.1.1^3,7^]decyl)benzaldehyde (9)^32^

To a stirring solution of Weinreb amide 8 (750 mg, 2.5 mmol) in THF (15 mL) under argon at 0 °C, LDBBA^36^ (17.1 mL, 0.44 M, 7.5 mmol) was added dropwise and the reaction mixture was stirred at 0 °C for 2 h. The reaction was quenched with an aqueous solution of sodium bicarbonate. The two phases were separated and the aqueous layer was extracted with EtOAc. The combined organic layers are dried over Na_2_SO_4_ and concentrated under vacuum to afford 9. The product was used without further purification to the next reaction.

##### 4-(1-Tricyclo[3.3.1.1^3,7^]decyl)-*N*-methoxy-*N*-methylbenzamide (8)^37^

###### A method

To a stirring solution of 4-(1-adamantyl)benzoic acid (7) (500 mg, 1.95 mmol) in dichloromethane (10 mL) under argon, at 0 °C, DIPEA (mmol 0.5 mL, 2.93 mmol) and Deoxo-Fluor (0.9 mL, 2.7 M, 2.34 mmol) was added. The reaction mixture was stirred at the same temperature for 30 min. To a solution of *N*,*O*-dimethylhydroxylamine hydrochloride (286 mg, 2.93 mmol) in dichloromethane (5 mL), DIPEA (0.5 mL) was added under cooling (∼0 °C) and the reaction mixture is stirred for 5 min. The prepared solution of *N*,*O*-dimethylhydroxylamine was added to the acyl fluoride mixture and stirred at 0 °C for 30 min, and then was heated to 55 °C for 24 h. An aqueous solution of NaHCO_3_ was added to the reaction mixture and the aqueous layer was extracted with dichloromethane. The organic layer was washed with brine, dried over Na_2_SO_4_ and concentrated under vacuum. The residue was purified by gradient flash column chromatography. Elution with a mixture of EtOAc : *n*-hexane : acetic acid, 1 : 10 : 0.01 to 3 : 7 : 0.01 afforded 303 mg of 8. Yield 52%.

###### B method

To a stirring solution of 4-(1-adamantyl)benzoic acid (7) (600 mg, 2.34 mmol) and PPh_3_ (920 mg, 3.51 mmol) in anhydrous dichloromethane (20 mL) at 0 °C, NBS (625 mg, 3.51 mmol) was added and the reaction mixture was stirred under cooling for 45 min. Then, the reaction mixture was stirred at rt and *N*,*O*-dimethylhydroxylamine hydrochloride (342 mg, 8.23 mmol) and triethylamine (0.35 mL, 2.54 mmol) were added and the resulting mixture was stirred at rt for 24 h. The reaction was quenched with an aqueous solution of sodium bicarbonate. The two phases were separated and the aqueous layer was extracted with EtOAc. The combined organic layers were dried over Na_2_SO_4_ and concentrated under vacuum. The residue was purified by flash column chromatography with an eluent mixture of 10% EtOAc in dichloromethane to afford 450 mg of 8. Yield 64%. M.p.: 123–124 °C.

##### α-Methylamino-[4-(1-tricyclo[3.3.1.1^3,7^]decyl)phenyl]acetonitrile hydrochloride (10)

To a stirring suspension of sodium cyanide (588 mg 12, mmol) and methylamine hydrochloride (810 mg, 12 mmol) in a mixture of DMSO : H_2_O, 9 : 1 (5 mL), a solution of carboxaldehyde 9 (1.44 g, 6 mmol) in DMSO (10 mL) was added and the reaction mixture was stirred under argon atmosphere at rt for 48 h and then was heated at 60 °C for 7 h. The reaction was quenched with an aqueous solution of sodium bicarbonate. The two phases were separated and the aqueous layer was extracted with diethyl ether. The combined organic layers were washed with water, dried over Na_2_SO_4_ and concentrated under vacuum. The residue was transformed into hydrochloride. 1.35 g, yield 80%. M.p.: 238–241 °C (ethanol-diethyl ether); ^1^H NMR (400 MHz, DMSO-*d*_6_) *δ* (ppm): 10.95 (br. s, 2H, NH_2_), 7.66–7.68 (d, 2H, *J* = 8.4 Hz, 2,6-Har), 7.51–7.53 (d, 2H, *J* = 8.4 Hz, 3,5-Har), 6.01 (s, 1H, α-H), 2.58 (s, 3H, CH_3_), 2.06 (br. s, 3H, 3,5,7-Had), 1.86–1.87 (m, 6H, 2,8,9-Had), 1.70–1.76 (m, 6H, 4,6,10-Had). ^13^C-NMR (101 MHz, DMSO-*d*_6_) *δ* (ppm): 170.7 (1-Car), 153.6 (4-Car), 129.2 (2,6-Car), 125.7 (3,5-Car), 115.2 (CN), 50.7 (α-C), 42.3 (2,8,9-Cad), 41.9 (1-Cad), 36.0 (4,6,10-Cad), 30.9 (CH_3_), 28.2 (3,5,7-Cad).

##### *N*′-Methyl-1-[4-(1-tricyclo[3.3.1.1^3,7^]decyl)phenyl]-1,2-ethane diamine dihydrochloride (11)

To a stirring solution of nitrile hydrochloride 10 (1.1 g 3.9 mmol) in ethanol (50 mL) and saturated ethanolic hydrogen solution (5 mL), Adam's catalyst platinum oxide (150 mg) was added into the reaction mixture, which then was hydrogenated under 55 psi for 4.5 h. The catalyst is then filtered off and the filtrate was concentrated under vacuum to afford the diamine dihydrochloride 1.1 g of 11. Almost quantitative yield. M.p.: >250 °C (methanol-ether); ^1^H-NMR (400 MHz, DMSO-*d*_6_) *δ* (ppm): 10.44 (br. s, 1H, NH), 9.99 (br. s, 1H, NH), 8.54 (br. s, 3H, NH_3_), 7.59–7.61 (d, 2H, *J* = 8.4 Hz, 2,6-Har), 7.46–7.48 (d, 2H, *J* = 8.4 Hz, 3,5-Har), 4.49–4.52 (m, 1H, α-H), 3.62–3.67 (m, 1H, β-H), 3.34–3.39 (m, 1H, β-H), 2.32 (s, 3H, CH_3_), 2.06 (s, 3H, 3,5,7-Had), 1.87 (s, 6H, 2,8,9-Had), 1.73–1.74 (m, 6H, 4,6,10-Had). ^13^C-NMR (101 MHz, DMSO-*d*_6_) *δ* (ppm): 152.4 (1,4-Car), 128.9 (2,6-Car), 125.5 (3,5-Car), 59.4 (α-C), 42.4 (2,8,9-C), 40.5 (β-C), 36.1 (4,6,10-Cad), 35.8 (1-Cad), 30.2 (CH_3_), 28.3 (3,5,7-Cad).

##### 4,5-Dihydro-1-methyl-5-[4-(1-tricyclo[3.3.1.1^3,7^]decyl)phenyl]-1*H*-imidazole (1a)

Diamine dihydrochloride 11 (370 mg, 1.3 mmol) was dissolved in absolute ethanol (10 mL) and formamidine acetate (177 mg, 1.7 mmol) is added and the reaction mixture was stirred at rt under argon atmosphere for 24 h and then was heated to 50 °C for 24 h. Then, the solvent was removed under vacuum and the residue was treated with solution HCl 4% (30 mL) and the resulting mixture was washed with EtOAc. The aqueous layer was basified with a solution of NaOH 4% and then extracted with EtOAc. The combined organic layers were washed with brine, dried over Na_2_SO_4_ and concentrated under vacuum to afford 227 mg of 1a, yield: 77%. The product was transformed into fumarate. M.p.: 202–204 °C (methanol-ether); ^1^H-NMR (400 MHz, DMSO-*d*_6_) *δ* (ppm): 8.10 (br. s, 1H, 2-Him), 7.40–7.43 (d, 2H, *J* = 8.2 Hz, 2,6-Har), 7.29–7.31 (d, 2H, *J* = 8.2 Hz, 3,5-Har), 6.48 (s, 2H, CH_2_=, fumarate), 4.85–4.90 (m, 1H, 5-Him), 4.22–4.28 (m, 1H, 4-Him), 3.57–3.63 (m, 1H, 4-Him), 2.78 (s, 3H, CH_3_), 2.05 (s, 3H, 3,5,7-Had), 1.85 (s, 6H, 2,8,9-Had), 1.73–1.74 (m, 6H, 4,6,10-Had). ^13^C-NMR (101 MHz, DMSO-*d*_6_) *δ* (ppm): 167.8 (C=O), 158.1 (2-Cim), 151.2 (1,4-Car), 135.1 (CH=, fumarate), 127.0 (2,6-Car), 125.4 (3,5-Car), 64.9 (5-Cim), 57.0 (4-Cim), 42.5 (2,8,9-Cad), 36.1 (4,6,10-Cad), 35.7 (1-Cad), 32.2 (CH_3_), 28.27 (3,5,7-Cad). Anal. calc. for C_24_H_30_N_2_O_4_ (%): C, 70.22; H, 7.37; N. 6.82, found: (%) C, 70.46; H, 7.01; N, 6.58.

##### 4,5-Dihydro-1,2-dimethyl-5-[4-(1-tricyclo[3.3.1.1^3,7^]decyl)phenyl]-1*H*-imidazole (1b)

To a solution of diamine dihydrochloride 11 (370 mg, 1.3 mmol) in absolute ethanol (10 mL) acetamidine hydrochloride (161 mg, 1.7 mmol) was added and the reaction mixture was stirred at rt under argon atmosphere for 24 h and then was heated to 50 °C for 24 h. Then, the solvent was removed under vacuum and the residue was treated with a solution of HCl (30 mL, 4%). The resulting mixture was washed with EtOAc and the aqueous layer was basified with solution NaOH 4% and extracted with EtOAc. The combined organic layers were washed with brine, dried over Na_2_SO_4_ and concentrated under vacuum to afford 201 mg of 1b, yield: 65%. The product was transformed into fumarate. M.p.: 182–184 °C (ethanol-diethyl ether); ^1^H-NMR (DMSO-*d*_6_) *δ* (ppm): ^1^H-NMR (400 MHz, DMSO-*d*_6_) *δ* (ppm): 7.42–7.44 (d, 2H, *J* = 8.4 Hz, 3,5-Har), 7.32–7.34 (d, 2H, *J* = 8.4 Hz, 2,6-Har), 6.45 (s, 2H, CH=, fumarate), 5.03–5.09 (m, 1H, 5-Him), 4.15–4.21 (m, 1H, 4-Him), 3.53–3.59 (m, 1H, 4-Him), 2.75 (s, 3H, 1-CH_3_), 2.32 (s, 3H, 2-CH_3_), 2.05 (s, 3H, 3,5,7-Had), 1.86 (s, 6H, 2,8,9-Had), 1.73–1.74 (m, 6H, 4,6,10-Had). ^13^C-NMR (101 MHz, DMSO-*d*_6_) *δ* (ppm): 168.1 (C=O), 166.9 (2-Cim), 151.7 (1,4-Car), 135.3 (CH=, fumarate), 127.2 (2,6-Car), 125.5 (3,5-Car), 65.6 (5-Cim), 52.1 (4-Cim), 42.5 (2,8,9-C), 36.1 (4,6,10-Cad), 35.7 (1-Cad), 30.8 (1-CH_3_), 28.3 (3,5,7-Cad), 11.9 (2-CH_3_). Anal. calc. for C_25_H_32_N_2_O_4_ (%): C, 70.73; H, 7.60; N, 6.60; found: (%) C, 70.26; H, 7.77; N, 6.39.

##### 4,5-Dihydro-1-methyl-5-[4-(1-tricyclo[3.3.1.1^3.7^]decyl)phenyl]-1*H*-2-imidazolamine (1c)

To a stirring solution of diamine 11 (825 mg, 2.9 mmol) in anhydrous dichloromethane (10 mL) at 0 °C, a solution of cyanogen bromide (384 mg, 3.6 mmol) in anhydrous dichloromethane (5 mL) was added dropwise and the reaction mixture was stirred at rt, under argon atmosphere for 48 h. Then, the reaction mixture was concentrated under vacuum and the residue was treated with anhydrous diethyl ether and the hydrobromide crystallized to afford 258 mg of 1c. Yield of hydrobromide: 66%. M.p.: 253–255 °C (dec). (ethanol-diethyl ether); ^1^H NMR (DMSO-*d*_6_) *δ* (ppm): 8.15 (s, 1H, 3-Him), 7.99 (s, 1H, 2-Him), 7.41–7.43 (d, 2H, *J* = 8.2 Hz, 2,6-Har), 7.28–7.30 (d, 2H, *J* = 8.2 Hz, 3,5-Har), 4.86–4.91 (m, 1H, 5-Him), 3.92–3.97 (m, 1H, 4-Him), 3.30–3.38 (m, 1H, 4-Him), 2.68 (s, 3H, CH_3_), 2.04 (s, 3H, 3,5,7-Had), 1.85 (s, 6H, 2,8,9-Had), 1.72 (m, 6H, 4,6,10-Had). ^13^C NMR (DMSO-*d*_6_) *δ* (ppm): 158.8 (2-Cim), 151.4 (1-Car), 134.8 (4-Car), 127.0 (3,5-Car), 125.4 (2,6-Car), 63.9 (5-Cim), 49.4 (4-Cim), 42.5 (2,8,9-Cad), 36.1 (4,6,10-Cad), 35.7 (1-Cad), 29.1 (CH_3_), 28.3 (3,5,7-Cad). Anal. calc. for C_20_H_28_BrN_3_ (%): C, 61.54; H, 7.23; N, 10.76, found: (%) C, 61.25; H, 7.42; N, 10.70.

##### 4,5-Dihydro-2-[4-(1-tricyclo[3.3.1.1^3.7^]decyl)phenyl]-1*H*-imidazole (1d)

To a stirring solution of aldehyde 9 (240 mg, 1 mmol) in *tert*-butyl alcohol (10 mL), ethylenediamine (66 mg, 1.1 mmol) was added and the reaction mixture was stirred at rt for 30 min. Subsequently, K_2_CO_3_ (417 mg, 3 mmol) and iodine (3.18 mg, 1.25 mmol) were added and the reaction mixture was heated to 70 °C for 3 h. In the case of complete discoloration, the excess iodine is destroyed by adding sodium metabisulphite under cooling. *Tert*-butanol is removed *in vacuo*, water is added to the residue and the aqueous layer is extracted with chloroform. The combined organic layers are washed with water, dried over Na_2_SO_4_ and concentrated under vacuum to afford 280 mg of 1d. Yield almost quantitative. M.p.: 161–163 °C. The product was transformed into fumarate. M.p.: >250 °C (ethanol-diethyl ether); ^1^H-NMR (400 MHz, CDCI_3_) *δ* (ppm): 7.73–7.75 (d, 2H, *J* = 8.4 Hz, 2,6-Har), 7.37–7.39 (d, 2H, *J* = 8.4 Hz, 3,5-Har), 5.04 (br.s, 1H, 1-Him), 3.78 (s, 4H, 4,5-Him), 2.09 (br. s, 3H, 3,5,7-Had), 1.89–1.90 (m, 6H, 2,8,9-Had), 1.72–1.81 (m, 6H, 4,6,10-Had). ^13^C-NMR (101 MHz, CDCl_3_) *δ* (ppm): 165.0 (2-Cim), 154.1 (1,4-Car), 127.1 (2,6-Car), 125.2 (3,5-Car), 50.0 (4,5-Cim), 43.1 (2,8,9-Cad), 36.8 (4,6,10-Cad), 36.6 (1-Cad), 29.0 (3,5,7-Cad). Anal. calcd. for C_23_H_28_N_2_O_4_ (%): C, 69.67; H, 7.12; N, 7.07; found (%) C, 69.40; H, 7.27; N, 6.88.

#### General procedure of preparation of hydroxybenzimidamides 12, 16, 19

A solution of hydroxylamine hydrochloride (4.53 mmol) and DIPEA (4.53 mmol) in EtOH (20 mL) was stirred at rt for 1 h. Then, a solution of the respective benzonitrile 12, 14, 19 (1.51 mmol) in EtOH (50 mL) was added to the reaction mixture. The reaction mixture was heated to 60 °C for 16 h. Then, the solvent was removed under vacuum, water was added to the reaction mixture and the aqueous phase was extracted with AcOEt. The combined organic phases were dried over Na_2_SO_4_ and concentrated under vacuum. The residue was purified by flash column chromatography with an eluent mixture of 20% methanol in dichloromethane to afford the respective benzimidamides as white solids.

##### (*Z*)-4-(1-Tricyclo[3.3.1.1^3,7^]decyl)-*N*-hydroxybenzimidamide (13)

*N*-Hydroxybenzimidamide 13 was prepared, as described in the general method, using benzonitrile 12. Yield: 78%. M.p.: 223–224 °C. ^1^H NMR (600 MHz, DMSO-*d*_6_), *δ* (ppm): 9.52 (s, 1H, OH), 7.60–7.59 (d, *J* = 8.5 Hz, 2H, 2,6-Had), 7.35–7.34 (d, *J* = 8.5 Hz, 2H, 3,5-Had), 5.72 (s, 2H, NH_2_), 2.06–2.05 (s, 3H, 3,5,7-Had), 1.86 (m, 6H, 2,8,9-Had), 1.74–1.73 (m, 6H, 4,6,10-Had). ^13^C NMR (151 MHz, DMSO-*d*_6_), *δ* (ppm): 151.4 (4-Car), 150.7 (N–C=N), 124.3 (1-Car), 125.1 (2,6-Car), 124.3 (3,5-Car), 42.3 (2,8,9-Cad), 36.0 (4,6,10-Cad), 35.6 (1-Cad), 28.1 (3,5,7-Cad).

##### 4-[4-(1-Tricyclo[3.3.1.1^3,7^]decyl)phenoxy]-*N*′-hydroxybenzimidamide (17)

*N*-Hydroxybenzimidamide 17 was prepared, as described in the general method, using benzonitrile 16. Yield: 71%. M.p.: 217–218 °C. There is a mixture of *E* and Z isomers, with the *Z* isomer 72% and the *E* isomer 28%. *Z* isomer: ^1^H NMR (400 MHz, DMSO-*d*_6_), *δ* (ppm): 9.54 (s, 1H, OH), 7.67–7.65 (d, *J* = 8.8 Hz, 2H, 2′,6′-Har), 7.38–7.36 (d, *J* = 8.8 Hz, 2H, 3,5-Har), 6.98–6.94 (m, 4H, 3′,5′,2,6-Har), 5.75 (s, 2H, NH2), 2.06–2.04 (br.s, 3H, 3,5,7-Had), 1.86–1.85 (m, 6H, 2,8,9-Had), 1.73 (m, 6H, 4,6,10-Had). ^13^C NMR (101 MHz, DMSO-*d*_6_), *δ* (ppm): 157.41 (4′-Car), 153.68 (1-Car), 150.15 (N–C=N), 146.17 (4-Car), 128.07 (1′-Car), 126.92 (2′,6′-Car), 126.10 (3,5-Car), 118.35 (2,6-Car), 117.74 (3′,5′-Car), 42.49 (2,8,9-Cad), 35.92 (4,6,10-Cad) – 35.20 (1-Cad), 28.10 (3,5,7-Cad). *E* isomer: ^1^H NMR (400 MHz, DMSO-*d*_6_), *δ* (ppm): 9.54 (s, 1H, OH), 7.89–7.86 (d, *J* = 8.7 Hz, 2H, 2′,6′-Har), 7.40–7.38 (d, *J* = 8.8 Hz, 2H, 3,5-Har), 7.02–6.97 (m, 4H, 3′,5′,2,6-Har), 5.75 (s, 2H, NH2), 2.06–2.04 (br.s, 3H, 3,5,7-Had), 1.86–1.85 (m, 6H, 2,8,9-Had), 1.73 (m, 6H, 4,6,10-Had). ^13^C NMR (101 MHz, DMSO-*d*_6_), *δ* (ppm): 167.0 (N–C=N), 159.5 (4′-Car), 153.1 (1-Car), 146.7 (4-Car), 129.4 (2′,6′-Car), 128.6 (1′-Car), 126.2 (3,5-Car), 118.9 (2,6-Car), 116.9 (3′,5′-Car), 42.5 (2,8,9-Cad), 35.9 (4,6,10-Cad) – 35.2 (1-Cad), 28.1 (3,5,7-Cad).

##### (*Z*)-4-{[4-(1-Tricyclo[3.3.1.1^3,7^]decyl)benzyl]oxy}-*N*′-hydroxybenzimidamide (20)

*N*-Hydroxybenzimidamide 20 was prepared, as described in the general method, using benzonitrile 19. Yield 76%. M.p.: 218–219 °C (methanol-diethyl ether); ^1^H NMR (600 MHz, DMSO-*d*_6_), *δ* (ppm): 9.45 (s, 1H, OH), 7.60–7.58 (d, *J* = 8.8 Hz, 2H, 2′,6′-Har), 7.37 (s, 4H, 2,3,5,6-Har), 6.69–6.98 (d, *J* = 8.8 Hz, 2H, 3′,5′-Har), 5.72 (s, 2H, NH_2_), 5.07 (s, 2H, α-H), 2.06 (br.s, 3H, 3,5,7-Had), 1.86–1.85 (m, 6H, 2,8,9-Had), 1.76–1.70 (m, 6H, 4,6,10-Had). ^13^C NMR (151 MHz, DMSO-*d*_6_), *δ* (ppm): 159.4 (4′-Car), 151.0 (N–C=N), 134.4 (4-Car), 129.7 (1-Car), 128.1 (2,6-Car), 127.1 (2′,6′-Car), 126.3 (1′-Car), 125.2 (3,5-Car), 114.7 (3′,5′-Car), 69.4 (α-C), 43.0 (2,8,9-Cad), 36.6 (4,6,10-Cad), 36.1 (1-Cad), 28.7 (3,5,7-Cad).

#### General procedure of preparation of benzimidamide 2a, 3a, 3b

To a solution of the respective *N*′-hydroxybenzimidamide 13, 17, 20 (0.27 mmol) in AcOH (6.75 mL) was added acetic anhydride (0.35 mmol) and the reaction mixture was stirred under argon at rt for 1.5 h. Zinc powder (4.05 gr-at) was then added and the reaction mixture was further stirred for 24 h under argon at rt. By the end of the reaction, the zinc was removed by filtration and washed with AcOH and methanol. The filtrate was concentrated and the resulting residue was dissolved in methanol, a solution of NaOH (10 mL, 5 M) was added (pH > 7) and the resulting solution was stirred for 1 h. Subsequently, the solvent was removed under vacuum. Water and AcOEt was added to the reaction mixture and the aqueous phase was extracted with AcOEt. The combined organic phases were washed with water, dried over Na_2_SO_4_ and concentrated under vacuum. The residue was purified by gradient flash column chromatography with an eluent of 5% to 20% methanol in dichloromethane to afford the respective benzimidamine as white crystal.

##### 4-(1-Tricyclo[3.3.1.1^3,7^]decyl)benzimidamide diacetate (2a)

Benzimidamide 2a was prepared, as described in the general method, using *N*′-hydroxybenzimidamide 13. Elution with methanol/dichloromethane/acetic acid, 94 : 5 : 1 afforded 2a as a white crystal solid. Yield: 56%. M.p.: >250 °C. ^1^H NMR (400 MHz, DMSO-*d*_6_), *δ* (ppm): 10.21 (s, 3H, NH_2_, NH), 7.74 (d, *J* = 8.4 Hz, 2H, 2,6-Har), 7.56–7.54 (d, *J* = 8.4 Hz, 2H, 3,5-Har), 2.09–2.06 (s, 3H, 3,5,7-Had), 1.89–1.88 (m, 6H, 2,8,9-Had), 1.76–1.70 (m, 12H, 4,6,10-Had, CH_3_). ^13^C NMR (101 MHz, DMSO-*d*_6_), *δ* (ppm): 177.1 (CO), 166.1 (N–C=N), 156.3 (4-Car), 127.6 (2,6-Car), 127.2 (1-Car), 125.5 (3,5-Car), 42.4 (2,8,9-Cad), 36.4 (1-Cad), 36.2 (4,6,10-Cad), 28.4 (3,5,7-Cad), 24.9 (CH_3_); Anal. calc. for C_21_H_30_N_2_O_4_ (%): C, 67.35; H, 8.08; N, 7.48, found: (%) C, 67.55; H, 8.38; N, 7.28.

##### 4-[4-(1-Tricyclo[3.3.1.1^3,7^]decyl)phenoxy]benzimidamide (3a)

Benzimidamide 3a was prepared, as described in the general method, using *N*′-hydroxybenzimidamide 17 and afforded as a white crystal solid. Yield: 48%. M.p.: 238–239 °C (methanol-diethyl ether); ^1^H NMR (400 MHz, CD_3_OD), *δ* (ppm): 7.74 (d, *J* = 9.2 Hz, 2H, 2′,6′-Har), 7.43 (d, *J* = 8.0 Hz, 2H, 3,5-Har), 7.02 (m, 4H, 2,6,3′,5′-Har), 2.12–2.11 (br.s, 3H, 3,5,7-Had), 1.97–1.96 (br.s, 6H, 2,8,9-Had), 1.85–1.82 (m, 6H, 4,6,10-Had). ^13^C NMR (101 MHz, CD_3_OD), *δ* (ppm): 167.8 (N–C=N), 163.3 (4′-Car), 155.1 (1-Car), 149.6 (4-Car), 130.6 (2′,6′-Car), 128.0 (3,5-Car), 121.1 (2,6-Car), 118.9 (3′,5′-Car), 44.9 (2,8,9-Cad), 38.3 (4,6,10-Cad), 37.5 (1-Cad), 30.9 (3,5,7-Cad); Anal. calc. for C_23_H_26_N_2_O (%): C, 79.73; H, 7.56; N, 8.09, found: (%) C, 79.93; H, 7.76; N, 8.19.

##### 4-{[4-(1-Tricyclo[3.3.1.1^3,7^]decyl)benzyl]oxy}benzimidamide (3b)

Benzimidamide 3b was prepared, as described in the general method, using *N*′-hydroxybenzimidamide 20. Yield: 49%. M.p.: 243–244 °C (dec). ^1^H NMR (400 MHz, DMSO-*d*_6_), *δ* (ppm): 9.10 (s, 3H, NH_2_, NH), 7.83 (d, *J* = 8.4 Hz, 2H, 2′,6′-Har), 7.39 (s, 4H, 2,3,5,6-Har), 7.22 (d, *J* = 8.5 Hz, 2H, 3′,5′-Har), 5.19 (s, 2H, α-H), 2.05 (br.s, 3H, 3,5,7-Had), 1.85 (br.s, 6H, 2,8,9-Had), 1.73 (br.s, 6H, 4,6,10-Had). ^13^C NMR (101 MHz, DMSO-*d*_6_), *δ* (ppm): 164.49 (NH_2_CNH), 162.5 (4′-Car), 150.6 (4-Car), 133.1 (1-Car), 130.0 (2′,6′-Car), 127.6 (2,6-Car), 124.6 (3,5-Car), 119.5 (1′-Car), 114.9 (3′,5′-Car), 69.3 (α-C), 42.4 (2,8,9-Cad), 35.9 (4,6,10-Cad), 35.5 (1-Cad), 28.1 (3,5,7-Cad); Anal. calc. for C_24_H_28_N_2_O (%): C, 79.96; H, 7.83; N, 7.77, found: (%) C, 80.26; H, 8.03; N, 7.97.

#### General procedure of preparation of benzylethers 16, 19

Sodium hydride (5.15 mmol, 60%) was added to a solution of the respective alcohol 15, 18 (1.03 mmol) in anhydrous DMF (5 mL) and is allowed to stir for 10–15 min. Subsequently, a solution of 4-fluorobenzonitrile (1.03 mmol) in anhydrous DMF (5 mL) was added into the reaction mixture which was heated to 100 °C for 24 h. The reaction was quenched with water and the aqueous layer was extracted with EtOAc. The combined organic layers were washed with water and dried over Na_2_SO_4_ and concentrated under vacuum. The residue was purified by flash column chromatography with an eluent mixture of 10% EtOAc in *n*-hexane to afford the respective benzylether as white crystal product.

##### 4-[4-(1-Tricyclo[3.3.1.1^3,7^]decyl)phenoxy]benzonitrile (16)

Benzonitrile 16 was prepared, as described in the general method, using phenol 15.^32^ Yield 66%. M.p.: 206–207 °C; ^1^H NMR (400 MHz, CDCl_3_), *δ* (ppm): 7.59–7.57 (d, *J* = 8.9 Hz, 2H, 2′,6′-Har), 7.40–7.38 (d, *J* = 8.8 Hz, 2H, 3,5-Har), 7.01 (d, *J* = 2.3 Hz, 2H, 2,6-Har), 6.99–6.98 (d, *J* = 2.3 Hz, 2H, 3′,5′-Har), 2.12 (br.s, 3H, 3,5,7-Had), 1.92 (m, 6H, 2,8,9-Had), 1.83–1.74 (m, 6H, 4,6,10-Had). ^13^C NMR (101 MHz, CDCl_3_), *δ* (ppm): 162.5 (4′-Car), 152.8 (1-Car), 148.9 (4-Car), 134.5 (2′,6′-Car), 127.1 (3,5-Car), 120.4 (2,6-Car), 119.4 (CN), 118.2 (3′,5′-Car), 105.9 (1′-Car), 43.8 (2,8,9-Had), 37.2 (4,6,10-Had), 36.5 (1-Had), 29.4 (3,5,7-Had).

##### 4-{[4-(1-Tricyclo[3.3.1.1^3,7^]decyl)benzyl]oxy}benzonitrile (19)

Benzonitrile 19 was prepared, as described in the general method, using methanol 18.^32^ Yield 62%. M.p.: 173–174 °C. ^1^H NMR (600 MHz, CDCl_3_), *δ* (ppm): 7.59–7.58 (d, *J* = 8.9 Hz, 2H, 2′,6′-Har), 7.41–7.40 (d, *J* = 6.3 Hz, 2H, 3,5-Har), 7.37–7.35 (d, *J* = 8.4 Hz, 2H, 2,6-Har), 7.03–7.01 (d, *J* = 8.9 Hz, 2H, 3′,5′-Har), 5.08 (s, 2H, α-H), 2.11 (br.s, 3H, 3,5,7-Had), 1.93–1.91 (m, 6H, 2,8,9-Had), 1.82–1.72 (m, 6H, 4,6,10-Had). ^13^C NMR (151 MHz, CDCl_3_), *δ* (ppm): 162.5 (4′-Car), 152.2 (4-Car), 134.4 (2′,6′-Car), 133.0 (1-Car), 127.9 (2,6-Car), 125.7 (3,5-Car), 119.6 (CN), 115.9 (1′-Car), 70.6 (α-C), 43.5 (2,8,9-Cad), 37.1 (4,6,10-Cad), 36.5 (1-Cad), 29.3 (3,5,7-Cad).

##### 2-[4-(1-Tricyclo[3.3.1.1^3,7^]decyl)phenyl]acetimidamide (2b)

To a stirring solution of ammonium chloride (42 mg, 0.79 mmol) in toluene (0.4 mL) at 0 °C, a solution of trimethylaluminum in toluene (0.79 mL, 1 M, 0.79 mmol) was added dropwise. The reaction mixture was stirred at rt for 2 h. Acetonitrile 14 (200 mg, 0.7 mmol) was added, and the resulting reaction mixture was heated to 80 °C for 19 h. The reaction mixture was cooled and the aluminum complex was decomposed by carefully pouring the solution into a slurry of silica gel in chloroform. The resulting mixture was stirred at rt for 10 min. Then, the silica gel was filtered off and washed with methanol. The combined filtrates were concentrated under vacuum. The residue was purified by flash column chromatography with an eluent mixture of 10% methanol in dichloromethane and afforded 97 mg of 2b as a white crystal solid. Yield: 36%. M.p.: 240–242 °C. ^1^H NMR (600 MHz, DMSO-*d*_6_), *δ* (ppm): 9.34–8.93 (br.d, 3H, NH_2_, NH), 7.40 (d, *J* = 7.8 Hz, 2H, 2,6-Had), 7.31 (d, *J* = 7.9 Hz, 2H, 3,5-Had), 3.68 (s, 2H, α-H), 2.02 (s, 3H, 3,5,7-Had), 1.82–1.70 (m, 12H, 2,8,9,4,6,10-Had). ^13^C NMR (151 MHz, DMSO-*d*_6_), *δ* (ppm): 169.7 (N–C=N), 150.4 (4-Car), 131.6 (1-Cad), 128.9 (2,6-Cad), 125.2 (3,5-Car), 42.9 (2,8,9-Cad), 37.2 (α-C), 36.4 (4, 6, 10-Cad), 35.8 (1-Cad), 28.5 (3,5,7-Cad); Anal. calc. for C_18_H_24_N_2_ (%): C, 80.55; H, 9.01; N, 10.44, found: (%) C, 80.85; H, 8.23; N, 8.07.

#### General procedure for the preparation of benzaldehydes 21–23

To a stirring solution of the respective phenol 15 (2.63 mmol) in DMF (10 mL), the corresponding 4-fluorobenzaldehyde (7.9 mmol) and K_2_CO_3_ (7.9 mmol) were added. The reaction mixture was heated at 160 °C for 24 h. Then, EtOAc and HCl solution (1N) were added to the reaction mixture and the aqueous layer was extracted with EtOAc. The combined organic layers were dried over Na_2_SO_4_ and concentrated under vacuum. The residue was purified by flash column chromatography with an eluent mixture of 10% EtOAc in *n*-hexane to afford a product, which was further treated with ice-cold hexane to give the desired products 21–23.

##### 4-[4-(1-Tricyclo[3.3.1.1^3,7^]decyl)phenoxy]benzaldehyde (21)

Benzaldehyde 21 was prepared, as described in the general procedure, using 4-fluorobenzaldehyde. Yield 55%. M.p.: 159 °C. ^1^H NMR (600 MHz, CDCl_3_) *δ* (ppm): 10.28 (s, 1H, CHO), 7.81 (d, *J* = 8.7 Hz, 1H, 5′-Har), 7.33 (d, *J* = 8.6 Hz, 2H, 3,5-Har), 6.94 (d, *J* = 8.6 Hz, 2H, 2,6-Har), 6.89 (d, *J* = 2.2 Hz, 1H, 3′-Har), 6.85 (dd, *J* = 8.7, 2.2 Hz, 1H, 6′-Har), 2.05 (s, 3H, 3,5,7-Had), 1.86–1.87 (m, 6H, 2,8,9-Had), 1.67–1.71 (m, 6H, 4,6,10-Had). ^13^C NMR (151 MHz, CDCl_3_) *δ* (ppm): 188.7 (CHO), 163.8 (1′-Car), 152.1 (4-Car), 148.9 (4′-Car), 139.8 (C-Cl), 131.2 (5′-Car), 127.2 (1-Car), 126.9 (3,5-Car), 120.2 (2,6-Car), 118.3 (3′-Car), 116.2 (6′-Car), 43.4 (2,8,9-Cad), 36.9 (4,6,10-Cad), 36.2 (1-Cad), 29.1 (3,5,7-Cad).

##### 4-[4-(1-Tricyclo[3.3.1.1^3,7^]decyl)phenoxy]-2-chlorobenzaldehyde (22)

Benzaldehyde 22 was prepared, as described in the general procedure, using 2-chloro-4-fluorobenzaldehyde. Yield 49%. M.p.: 165–167 °C. ^1^H NMR (600 MHz, CDCl_3_) *δ* 11.35 (s, 1H, CHO), 7.88 (d, *J* = 8.7 Hz, 1H, 5′-Har), 7.40 (d, *J* = 8.6 Hz, 2H, 3,5-Har), 7.02 (d, *J* = 8.6 Hz, 2H, 2,6-Har), 6.96 (d, *J* = 2.2 Hz, 1H, 3′-Har), 6.92 (dd, *J* = 8.7, 2.2 Hz, 1H, 6′-Har), 2.13 (s, 3H, 3,5,7-Had), 1.93 (m, 6H, 2,8,9-Had), 1.75–1.83 (m, 6H, 4,6,10-Had). ^13^C NMR (151 MHz, CDCl_3_) *δ* 188.7 (CHO), 163.8 (1′-Car), 152.1 (4-Car), 148.9 (4′-Car), 139.8 (C-Cl), 131.2 (5′-Car), 127.2 (1-Car), 126.9 (3,5-Car), 120.2 (2,6-Car), 118.3 (3′-Car), 116.1 (6′-Car), 43.4 (2,8,9-Cad), 36.8 (4,6,10-Cad), 36.2 (1-Cad), 29.1 (3,5,7-Cad).

##### 4-[4-(1-Tricyclo[3.3.1.1^3,7^]decyl)phenoxy]-3-chlorobenzaldehyde (23)

Benzaldehyde 23 was prepared, as described in the general procedure, using 3-chloro-4-fluorobenzaldehyde. Yield 74%. M.p.: 167–168 °C. ^1^H NMR (600 MHz, CDCl_3_) *δ* (ppm): 9.88 (s, 1H, CHO), 7.98 (d, *J* = 2.0 Hz, 1H, 2′-Har), 7.66 (dd, *J* = 8.5, 2.0 Hz, 1H, 6′-Har), 7.41–7.39 (m, 2H, 3′,5′-Har), 7.03–7.01 (m, 2H, 2,6-Har), 6.93 (d, *J* = 8.5 Hz, 1H, 5′-Har), 2.11 (s, 3H, 3,5,7-Had), 1.93–1.92 (m, 6H, 2,8,9-Had), 1.82–1.74 (m, 6H, 4,6,10-Had). ^13^C NMR (151 MHz, CDCl_3_) *δ* (ppm): 189.8 (CHO), 159.1 (1′-Car), 152.6 (1-Car), 148.7 (4-Car), 132.1 (2′-Car), 131.9 (Cl-C), 129.8 (6′-Car), 126.8 (3,5-Car), 125.2 (4′-Car), 119.7 (2,6-Car), 117.6 (5′-Car), 43.4 (2,8,9-Cad), 36.8 (4,6,10-Cad), 36.2 (1-Cad), 29.0 (3,5,7-Cad).

#### General procedure for the preparation of aminoguanidine hydrazones 4a, 5a–c

To a stirring solution of the respective benzaldehyde 9, 21–23 (0.42 mmol) and aminoguanide bicarbonate (0.42 mmol) in EtOH (5 mL), con. HCl (six drops) were added and the reaction mixture was heated to reflux for 24 h. Then, the solvent was evaporated and chloroform was added to the solid residue. The resulting mixture was filtered. The filtrate was concentrated under vacuum and the product was crystallised by anhydrous diethyl ether.

##### 2-(*E*)-4-[(1-Tricyclo[3.3.1.1^3,7^]decyl)benzylidene]hydrazine-1-carboxyimidamide hydrochloride (4a)

Aminoguanidine hydrazone 4a was prepared as described in the general procedure using benzaldehyde 9. Yield 46%. M.p.: >250 °C; ^1^H-NMR (400 MHz, DMSO*-d*_6_) *δ* (ppm): 11.74 (s, 1H, =N–NH), 8.13 (s, 1H, α-H), 7.78 (d, *J* = 8.4 Hz, 2H, 2,6-Har), 7.68 (br.s 4H, NH_2_), 7.43 (d, *J* = 8.4 Hz, 2H, 3,5-Har), 2.06 (s, 3H, 3,5,7-Had), 1.87–1.88 (m, 6H, 2,8,9-H), 1.78–1.74 (m, 6H, 4,6,10-Had). ^13^C-NMR (101 MHz, DMSO-*d*_6_) *δ* (ppm): 155.0 (C=NH), 153.3 (1-Car), 146.8 (α-C), 130.5 (4-Car), 127.3 (2,6-Car), 124.8 (3,5-Car), 42.1 (2,8,9-Cad), 35.9 (1,6-Cad), 35.8 (4,10-Cad), 28.0 (3,5,7-Cad); Anal. calc. for C_18_H_25_ClN_4_ (%): C, 64.95; H, 7.57; N, 16.83, found: (%) C, 65.25; H, 7.77; N, 7.87.

##### 2-{(*E*)-4-[4-(1-Tricyclo[3.3.1.1^3,7^]decyl)phenoxy]benzylidene}hydrazine-1-carboxyimidamide hydrochloride (5a)

Aminoguanidine hydrazone 5a was prepared as described in general procedure using benzaldehyde 21. Yield 88%. M.p.: >250 °C (ethanol-diethyl ether); ^1^H NMR (600 MHz, DMSO-*d*_6_) *δ* (ppm): 12.04 (br.s, 1H, =NH–C), 8.15 (s, 1H, α-H), 7.86 (d, *J* = 8.5 Hz, 2H, 2′,6′-Har), 7.76 (br. s, 4H, NH_2_), 7.39 (d, *J* = 8.5 Hz, 2H), 6.99–7.02 (m, 4H, 2,6,3′,5′-Har), 2.05 (s, 3H, 3,5,7-Had), 1.86–1.87 (m, 6H, 2,8,9-Had), 1.71–1.73 (m, 6H, 4,6,10-Had). ^13^C NMR (151 MHz, DMSO-*d*_6_) *δ* (ppm): 159.0 (C=NH), 155.4 (4′-Car), 153.4 (1-Car), 146.8 (4-Car), 146.1 (α-C), 129.5 (2′,6′-Car), 128.3 (1′-Car), 126.4 (3,5-Car), 118.8 (2,6-Car), 118.0 (3′,5′-Car), 42.7 (2,8,9-Cad), 36.1 (4,6,10-Cad), 35.4 (1-Cad), 28.3 (3,5,7-Cad); Anal. calc. for C_24_H_29_ClN_4_O (%): C, 67.83; H, 6.88; N, 13.18, found: (%) C, 68.13; H, 7.08; N, 13.48.

##### 2-{(*E*)-4-[4-(1-Tricyclo[3.3.1.1^3,7^]decyl)phenoxy]-2-chlorobenzylidene}hydrazine-1-carboxyimidamide hydrochloride (5b)

Aminoguanidine hydrazone 5b was prepared as described in general procedure using benzaldehyde 22. Yield 83%. M.p.: >250 °C (ethanol-diethyl ether); ^1^H NMR (600 MHz, DMSO-*d*_6_) *δ* (ppm): 12.23 (s, 1H, =NH–), 8.49 (s, 1H, α-H), 8.31 (d, *J* = 8.8 Hz, 1H, 5′-Har), 7.86 (br.s, 4H, NH_2_), 7.44 (dd, *J* = 10.5 Hz, *J* = 8.2 Hz, 2H, 2,6-Har), 7.06–7.01 (m, 4H, 3,5,3′,6′-Har), 2.06 (s, 3H, 3,5,7-Had), 1.87 (m, 6H, 2,8,9-Had), 1.71–1.73 (m, 6H, 4,6,10-Had). ^13^C NMR (151 MHz, DMSO-*d*_6_) *δ* (ppm): 159.6 (C=NH), 155.3 (1-Car), 152.7 (4′-Car), 147.4 (4-Car), 142.2 (α-C), 134.3 (C-Cl), 129.3 (5′-Car), 126.6 (2,6-Car), 125.4 (1′-Car), 119.2 (3,5-Car), 118.0 (6′-Car), 117.3 (3′-Car), 42.6 (2,8,9-Cad), 36.1 (4,6,10-Cad), 35.5 (1-Cad), 28.3 (3,5,7-Cad); Anal. calc. for C_24_H_28_Cl_2_N_4_O (%): C, 62.75; H, 6.14; N, 12.20, found: (%) C, 63.05; H, 6.24; N, 12.10.

##### 2-{(*E*)-4-[4-(1-Tricyclo[3.3.1.1^3,7^]decyl)phenoxy]-3-chlorobenzylidene}hydrazine-1-carboxyimidamide hydrochloride (5c)

Aminoguanidine hydrazone 5c was prepared as described in general procedure using benzaldehyde 23. Yield 68%. M.p.: >250 °C (ethanol-diethyl ether); ^1^H NMR (600 MHz, DMSO*-d*_6_) *δ* (ppm): 11.97 (s, 1H, =N–NH–), 8.26 (d, *J* = 1.9 Hz, 1H, 2′-Har), 8.14 (s, 1H, 6′-Har), 8.23–7.43 (br. s, 4H, NH_2_), 7.71 (d, *J* = 1.9 Hz, 2H, 3,5-Har), 7.38 (m, 1H, 5′-Har), 6.95–7.02 (m, 2H, 2,6-Har), 2.06 (s, 3H, 3,5,7-Had), 1.85 (m, 6H, 2,8,9-Had), 1.70–1.72 (m, 6H, 4,6,10-Had). ^13^C NMR (151 MHz, DMSO-*d*_6_) *δ* (ppm): 155.4 (C=NH), 153.6 (1-Car), 146.8 (4-Car), 144.9 (2′-Car), 130.3 (1′-Car), 128.7 (6′-Car, α-C), 126.5 (3,5-Car), 124.8 (C-Cl), 119.9 (5′-Car), 117.8 (2,6-Car), 42.7 (2,8,9-Cad), 36.1 (4,6,10-Cad), 35.5 (1-Cad), 28.3 (3,5,7-Cad); Anal. calc. for C_24_H_28_Cl_2_N_4_O (%): C, 62.75; H, 6.14; N, 12.20, found: (%) C, 63.95; H, 6.34; N, 12.00.

##### 2-{(*E*)-4-(1-(Tricyclo[3.3.1.1^3,7^]decyl))benzylidene}hydrazine-1-carboxamide hydrochloride (4b)

To a stirring solution of benzaldehyde 9 (250 mg, 1.04 mmol) and semicarbazide hydrochloride (116 mg, 1.04 mmol) in EtOH (1 mL), conc. HCl (six drops) were added and the reaction mixture was heated to reflux for 6 h and left at rt overnight. Subsequently, the solvent is evaporated and anhydrous diethyl ether was added to the solid residue forming crystals, 160 mg, yield 46%. M.p.: >250 °C (methanol/chloroform/propanol); ^1^H NMR (400 MHz, DMSO-*d*_6_) *δ* (ppm): 10.17 (s, 1H, =N–NH), 7.80 (s, 1H, α-H), 7.62 (d, *J* = 8.5 Hz, 2H, 2,6-Har), 7.35 (d, *J* = 8.5 Hz, 2H, 3,5-Har), 6.43 (br.s, 2H, NH_2_), 2.05 (s, 3H, 3,5,7-Had), 1.86–1.87 (m, 6H, 2,8,9-Had), 1.73–1.74 (m, 6H, 4,6,10-Had). ^13^C NMR (101 MHz, DMSO*-d*_6_) *δ* (ppm): 156.7 (C=O), 151.9 (1-Car), 139.2 (α-C), 132.1 (4-Car), 126.4 (2,6-Car), 124.9 (4-Car), 42.4 (2,8,9-Cad), 36.1 (4,6,10-Cad), 35.8 (1-Cad), 28.3 (3,5,7-Cad); Anal. calc. for C_18_H_24_ClN_3_O (%): C, 64.76; H, 7.25; N, 12.59, found: (%) C, 63.96; H, 7.55; N, 12.49.

#### General procedure for the preparation of thiosemicarbazides 4c, 6a–c

To a stirring solution of the respective benzaldehyde 9, 21–23 (0.3 mmol) and thiosemicarbazide (0.3 mmol) in EtOH (10 mL), con. HCl (six drops) were added and the reaction mixture was heated to reflux for 24 h. Then, the solvent was concentrated under vacuum forming a crystal precipitate that was recrystallized by a mixture of solvents dichloromethane-EtOH, affording the pure product.

##### 2-(*E*)-4-[(1-Tricyclo[3.3.1.1^3,7^]decyl)benzylidene]hydrazine-1-carbothiamide hydrochloride (4c)

Thiosemicarbazide 4c was prepared as described in the general procedure. Yield 33%. M.p.: >250 °C (chloroform-EtOH); ^1^H NMR (600 MHz, DMSO*-d*_6_) *δ* 11.37 (s, 1H, =N–NH), 8.16 (s, 1H, NH), 8.02 (s, 1H, α-H), 7.92 (s, 1H, NH), 7.72 (d, *J* = 8.4 Hz, 2H, 2,6-Har), 7.39 (d, *J* = 8.4 Hz, 2H, 3,5-Har), 2.06 (s, 3H, 3,5,7-Had), 1.86–1.87 (m, 6H, 2,8,9-Had), 1.73–1.74 (m, 6H, 4,6,10-Had). ^13^C NMR (151 MHz, DMSO*-d*_6_) *δ* 177.8 (C=S), 152.8 (1-Car), 142.3 (α-C), 131.5 (4-Car), 127.2 (2,6-Car), 125.0 (3,5-Car), 42.4 (2,8,9-Cad), 36.1 (4,6,10-Cad), 35.9 (1-Cad), 28.2 (3,5,7-Cad); Anal. calc. for C_18_H_24_ClN_3_S (%): C, 61.78; H, 6.91; N, 12.01, found: (%) C, 62.08; H, 7.11; N, 12.31.

##### 2-{(*E*)-4-[4-(1-Tricyclo[3.3.1.1^3,7^]decyl)phenoxy]benzylidene}hydrazine-1-carbothioamide hydrochloride (6a)

Thiosemicarbazide 6a was prepared as described in the general procedure using benzaldehyde 21. Yield 52%. M.p.: >250 °C (dichloromethane-ethanol); ^1^H NMR (600 MHz, DMSO-*d*_6_) *δ* (ppm): 11.46 (s, 1H, =N–NH–), 8.23 (s, 1H, NH), 8.10 (s, 1H, α-H), 8.02 (s, 1H, NH), 7.88 (d, *J* = 8.7 Hz, 2H, 2′,6′-Har), 7.48 (d, *J* = 8.8 Hz, 2H, 3′,5′-Har), 7.07 (dd, *J* = 16.4, 8.8 Hz, 4H, 2,6,3′,5′-Har), 2.14 (s, 3H, 3,5,7-Had), 1.94–1.95 (m, 6H, 2,8,9-Had), 1.81–1.82 (m, 6H, 4,6,10-Had). ^13^C NMR (151 MHz, DMSO-*d*_6_) *δ* (ppm): 177.8 (C=S), 158.6 (4′-Car), 153.5 (1-Car), 146.7 (4-Car), 141.6 (α-C), 129.1 (2′,6′-Car), 129.1 (1′-Car), 126.4 (3,5-Car), 118.8 (2,6-Car), 118.0 (3′,5′-Car), 42.7 (2,8,9-Cad), 36.1 (4,6,10-Cad), 35.5 (1-Cad), 28.3 (3,5,7-Cad); Anal. calc. for C_24_H_28_ClN_3_OS (%): C, 65.22; H, 6.39; N, 9.51, found: (%) C, 65.52; H, 6.59; N, 9.61.

##### 2-{(*E*)-4-(4-(1-Tricyclo[3.3.1.1^3,7^]decyl)phenoxy)-2-chlorobenzylidene}hydrazine-1-carbothioamide hydrochloride (6b)

Thiosemicarbazide 6b was prepared as described in the general procedure using benzaldehyde 22. Yield 81%. M.p.: >250 °C (dichloromethane-ethanol); ^1^H NMR (400 MHz, DMSO-*d*_6_) *δ* (ppm): 13.12 (s, =N–NH–), 8.37 (s, 1H, α-H), 7.88–7.91 (m, 1H, 5′-Har), 7.51 (s, 2H, NH_2_), 7.27–7.31 (m, 2H, 2,6-Har), 6.88–6.92 (m, 2H, 3,5-Har), 6.80–6.83 (m, 2H, 3′,6′-Har), 2.03 (s, 3H, 3,5,7-Had), 1.83 (m, 6H, 2,8,9-Had), 1.69–1.71 (m, 6H, 4,6,10-Had). ^13^C NMR (101 MHz, DMSO-*d*_6_) *δ* (ppm): 177.8 (C=S), 158.9 (1-Car), 152.0 (4′-Car), 147.0 (4-Car), 138.8 (α-C), 134.3 (C-Cl), 127.5 (5′-Car), 125.7 (2,6-Car), 125.1 (1′-Car), 118.6 (3,5-Car), 117.4 (6′-Car), 116.0 (3′-Car), 42.4 (2,8,9-Cad), 35.8 (4,6,10-Cad), 35.0 (1-Cad), 27.9 (3,5,7-Cad); Anal. calc. for C_24_H_27_Cl_2_N_3_OS (%): C, 60.50; H, 5.71; N, 8.82, found: (%) C, 60.80; H, 5.81; N, 9.02.

##### 2-{(*E*)-4-[4-(1-Tricyclo[3.3.1.1^3,7^]decyl)phenoxy]-3-chlorobenzylidene}hydrazine-1-carbothioamide hydrochloride (6c)

Thiosemicarbazide 6c was prepared as described in general procedure using benzaldehyde 23. Yield 72%. M.p.: >250 °C (dichloromethane-ethanol); ^1^H NMR (400 MHz, CDCl_3_ + DMSO-*d*_6_) *δ* (ppm): 11.41 (=N–NH), 8.14 (s, 1H, NH), 8.13 (s, 1H, 2′-Har), 8.05 (s, 1H, NH), 7.97 (s, 1H, α-H), 7.55 (d, *J* = 8.0 Hz, 1H, 6′-Har), 7.30–7.33 (m, 2H, 3,5-Har), 6.90–6.93 (m, 3H, 2,6.5′-Har), 2.05 (s, 3H, 3,5,7-Had), 1.85 (m, 6H, 2,8,9-Had), 1.71–1.72 (m, 6H, 4,6,10-Had). ^13^C NMR (101 MHz, CDCl_3_ + DMSO-*d*_6_) *δ* (ppm): 178.0 (C=S), 153.50 (1-Car), 153.1 (4′-Car), 146.5 (4-Car), 140.0 (α-C), 130.9 (1′-Car), 128.2 (2′-Car), 127.90 (6′-Car), 126.20 (3,5-Car), 124.8 (C-Cl), 119.5 (5′-Car), 117.6 (2,6-Car), 42.6 (2,8,9-Cad), 36.1 (4,10-Cad), 35.3 (1,6-Cad), 28.2 (3,5,7-Cad); Anal. calc. for C_24_H_27_Cl_2_N_3_OS (%): C, 60.50; H, 5.71; N, 8.82, found: (%) C, 60.70; H, 5.51; N, 8.92.

## Data availability

The data supporting this article have been included into the ESI† and the experimental part of the manuscript.

## Conflicts of interest

There are no conflicts to declare.

## Supplementary Material

MD-OLF-D5MD00135H-s001
