# Supplementary material for: New Trypanosoma brucei acting derivatives incorporating 1-(4-phenyl)adamantane and 1-(4-phenoxyphenyl)adamantane
Source: RSC Med Chem. 2025 Apr 25;16(6):2441–51. doi: 10.1039/d5md00135h (PMC12053444; doi:10.1039/d5md00135h)
Supplement: MD-016-D5MD00135H-s001 [file MD-016-D5MD00135H-s001.pdf]

Supplementary Information (SI) for RSC Medicinal Chemistry.
This journal is © The Royal Society of Chemistry 2025

New *Trypanosoma brucei* acting derivatives incorporating 1-(4-phenyl)adamantane and 1-(4-phenoxyphenyl)adamantane

Konstantina Stavropoulou,[a]† Angeliki Kaimaki,[a]† Maria Nikolaou,[a]† Ana K. Brown,[a,b] Andrew Tsotinis,[a] Martin C. Taylor,[c] John M. Kelly [c] and Ioannis P. Papanastasiou*[a]

[a] Division of Pharmaceutical Chemistry, Department of Pharmacy, School of Health Sciences, National and Kapodistrian University of Athens, Panepistimiopoli-Zografou, 157 71 Athens, Greece.

[b] Department of Chemistry, University of San Francisco, 2130 Fulton Street, San Francisco, California, CA 94117, USA

[c] Department of Pathogen Molecular Biology, London School of Hygiene and Tropical Medicine, Keppel Street, London WC1 E7HT, UK.

† Equal contribution as co-first authors.

Table of Contents:
NMR SPECTRA

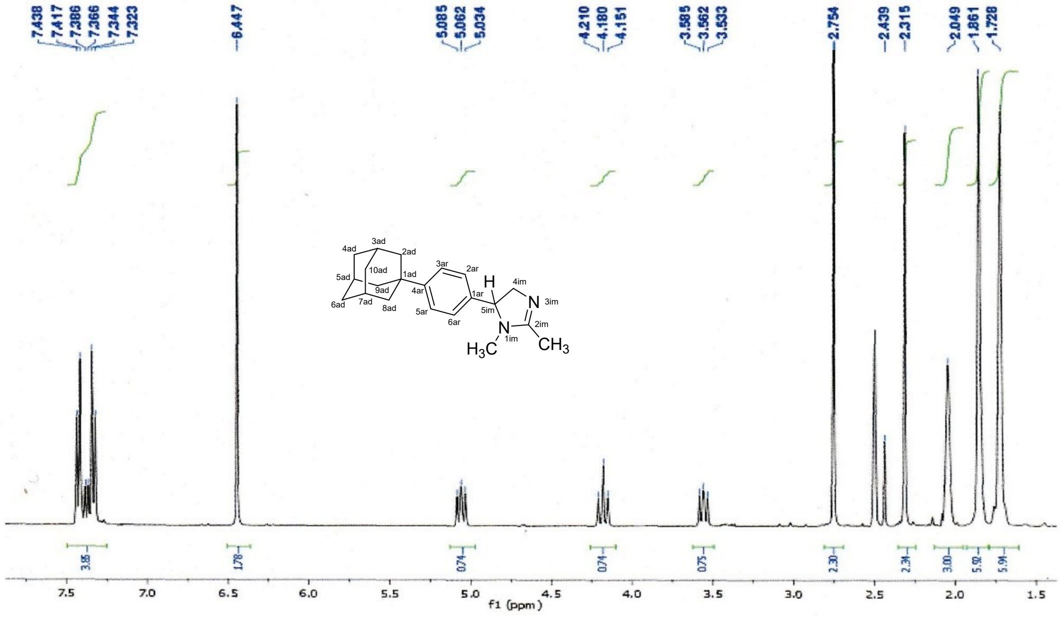

^{1}H NMR (DMSO-d_6) of 4,5-dihydro-1,2-dimethyl-5-[4-(1-tricyclo[3.3.1.1^{3,7}]decyl)phenyl]-1*H*-imidazole (1b).

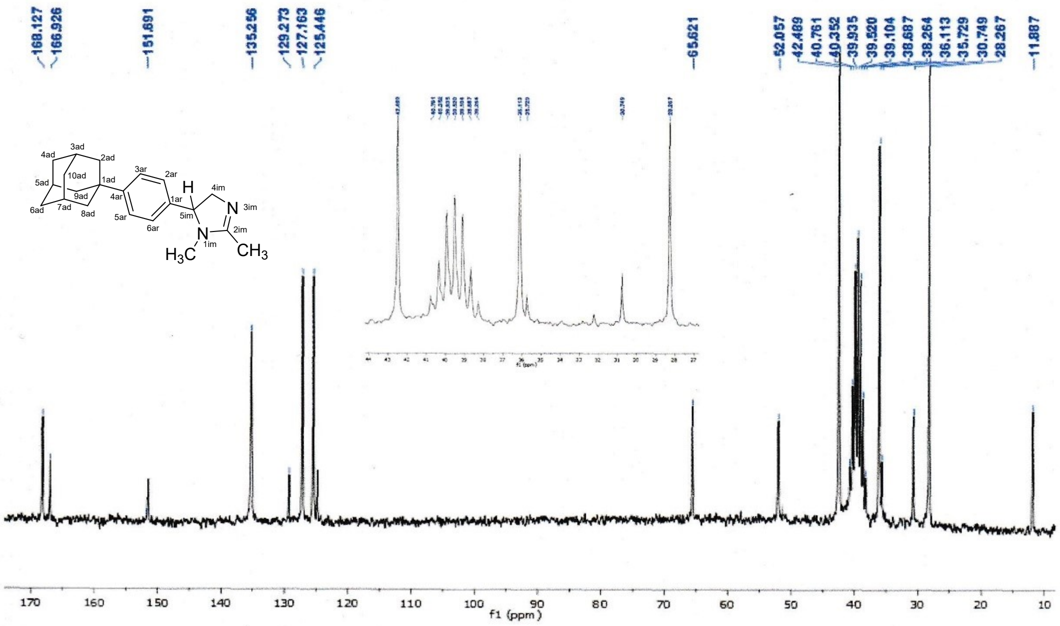

^{13}C NMR (DMSO- d_6) of 4,5-dihydro-1,2-dimethyl-5-[4-(1-tricyclo[3.3.1.1^{3,7}]decyl)phenyl]-1*H*-imidazole (1b).

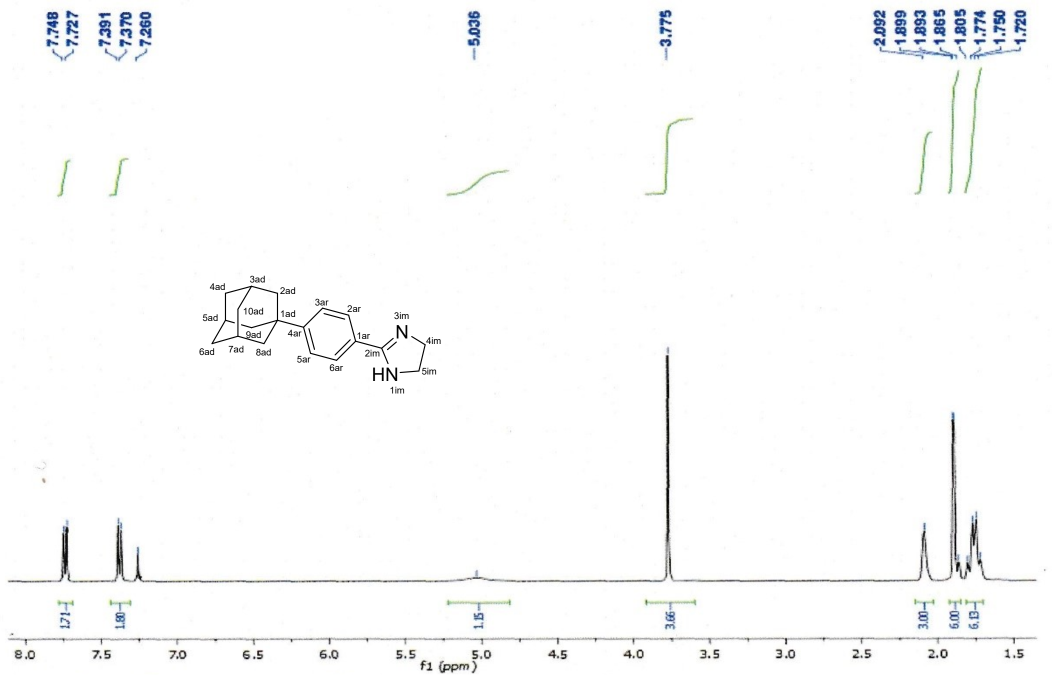

^{1}H NMR ($CDCl_3$) of 4,5-dihydro-2-[4-(1-tricyclo[3.3.1.1$^{3.7}$]decyl)phenyl]-1*H*-imidazole (1d).

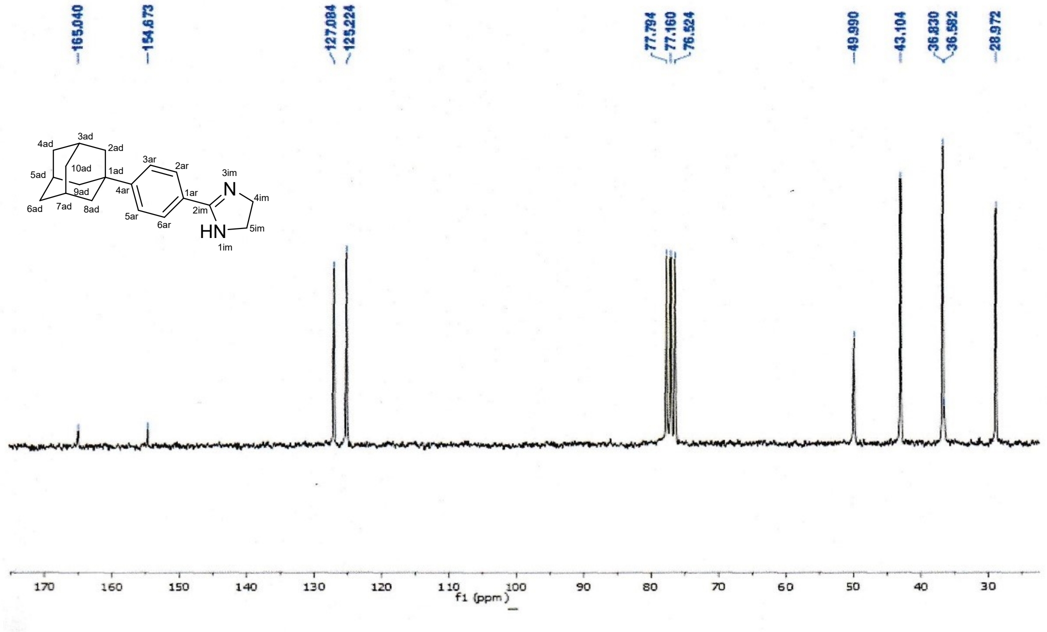

^{13}C NMR ($CDCl_3$) of 4,5-dihydro-2-[4-(1-tricyclo[3.3.1.1$^{3.7}$]decyl)phenyl]-1*H*-imidazole (1d).

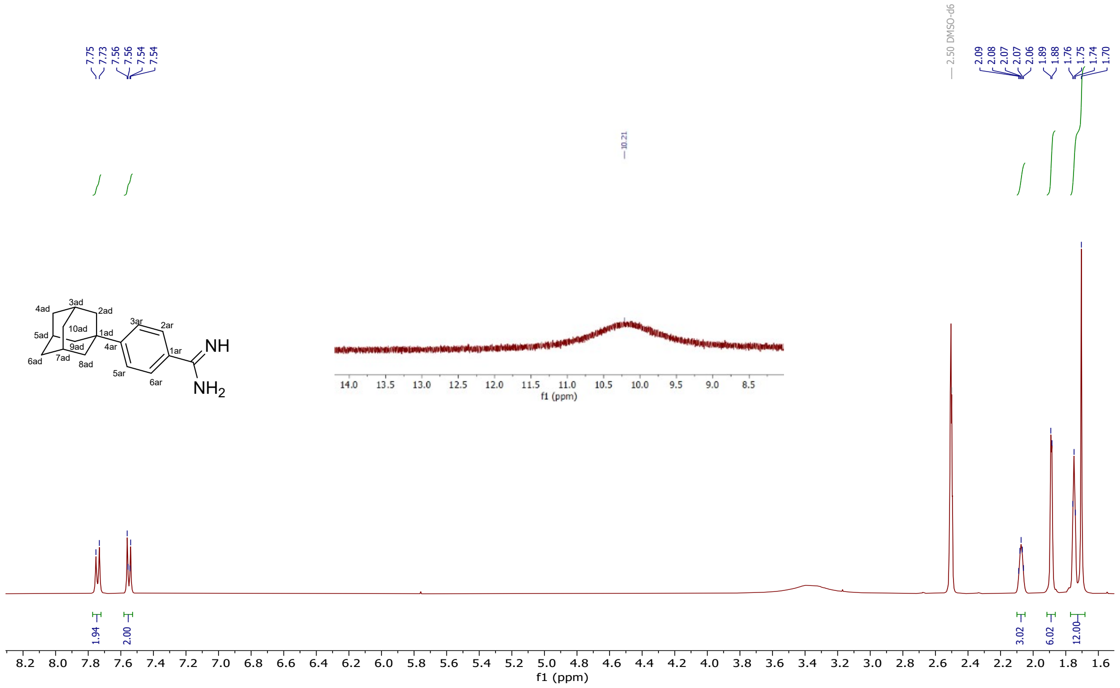

^1H NMR (400 MHz, DMSO-d_6) of 4-(tricyclo[3.3.1.1^{3,7}]dec-1-yl)benzimidamide diacetate (2a).

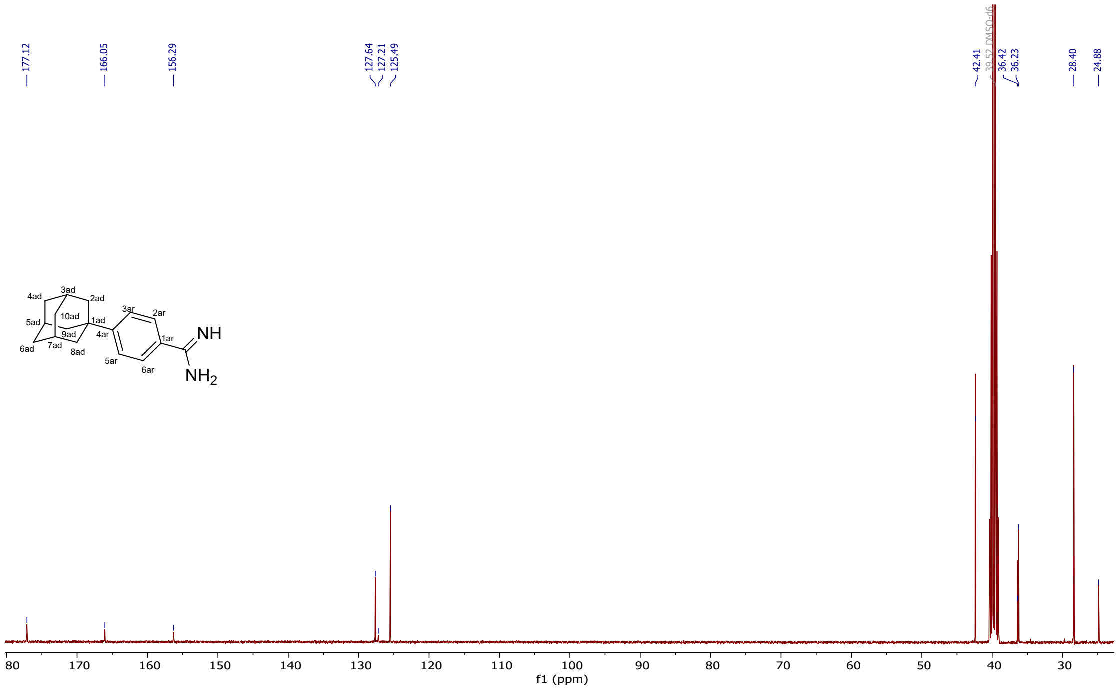

^{13}C NMR (101 MHz, DMSO-d_6) of 4-(tricyclo[3.3.1.1^{3,7}]dec-1-yl)benzimidamide diacetate (2a).

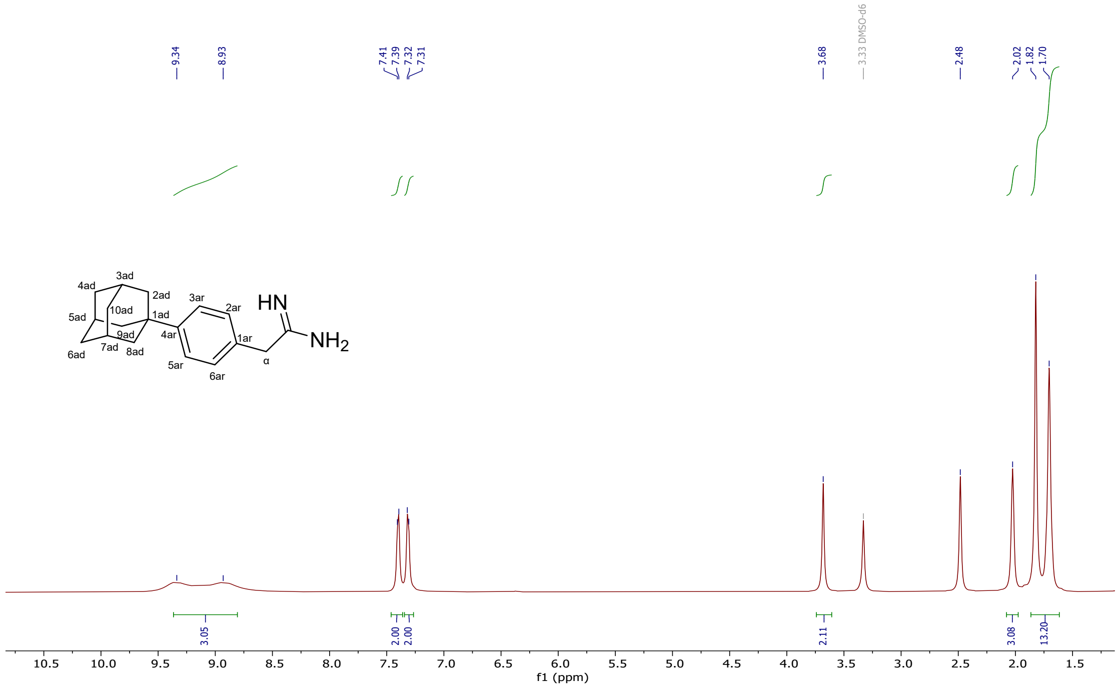

^{1}H NMR (600 MHz, DMSO-d_6) of 2-[4-(1-tricyclo[3.3.1.1^{3,7}]decyl)phenyl]acetimidamide (2b).

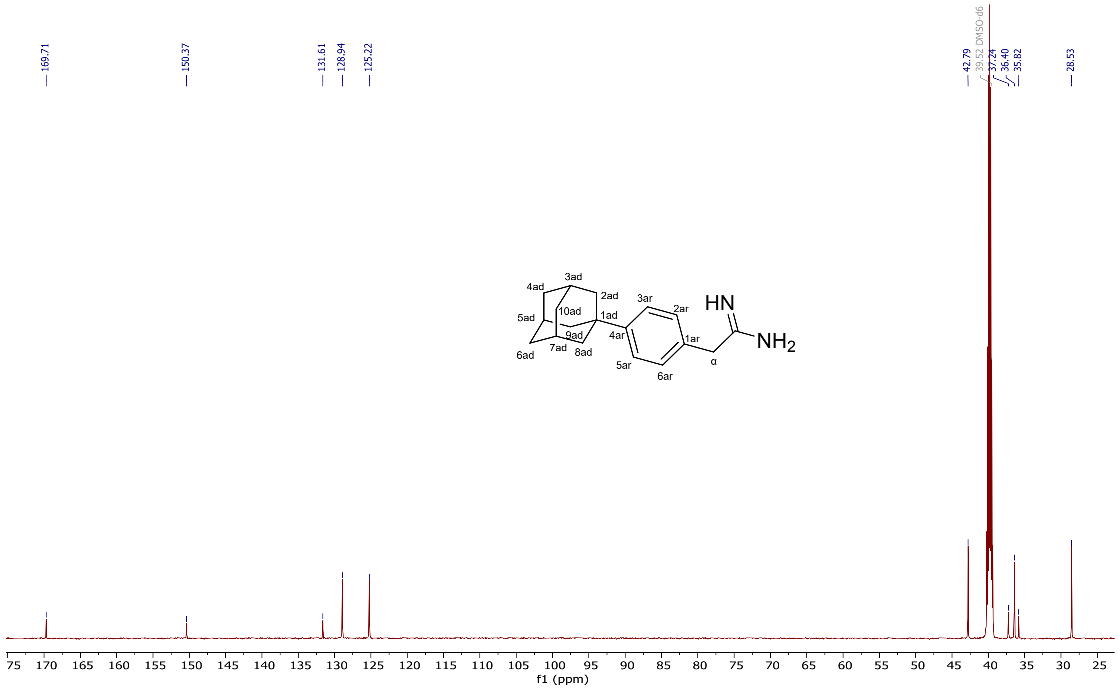

[13] C NMR (151 MHz, DMSO-d_6) of 2-[4-(1-tricyclo[3.3.1.1^{3,7}]decyl)phenyl]acetimidamide (2b).

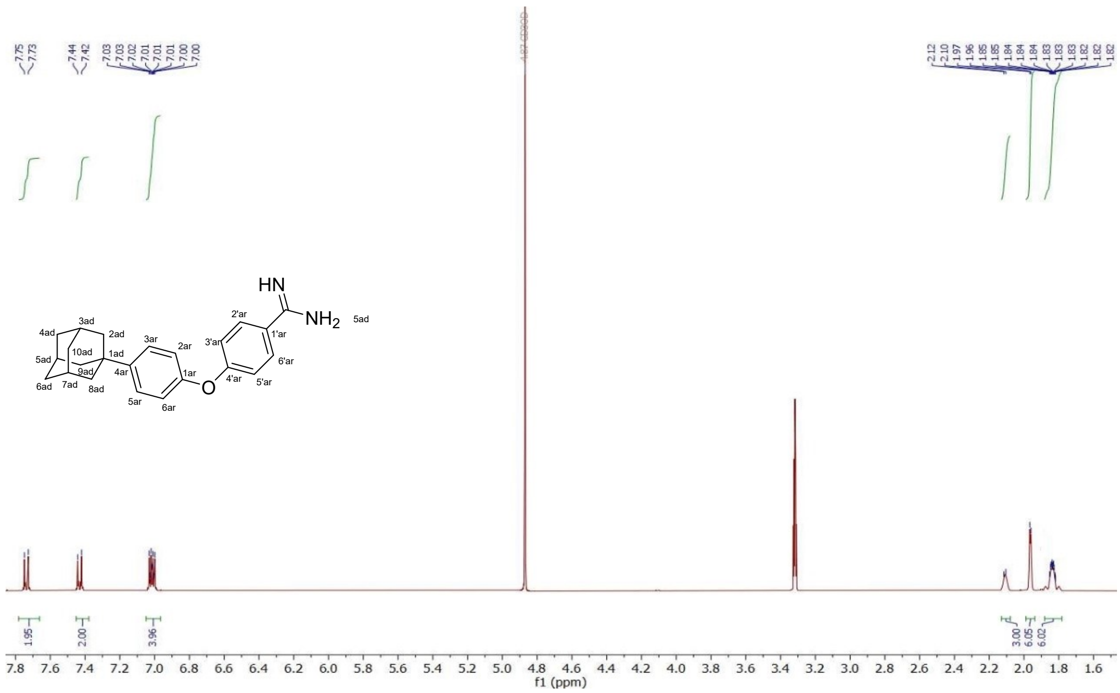

^{1}H NMR (400 MHz, CD_3OD) of 4-[4-(1-tricyclo[3.3.1.1^{3,7}]decyl)phenoxy]benzimidamide (3a).

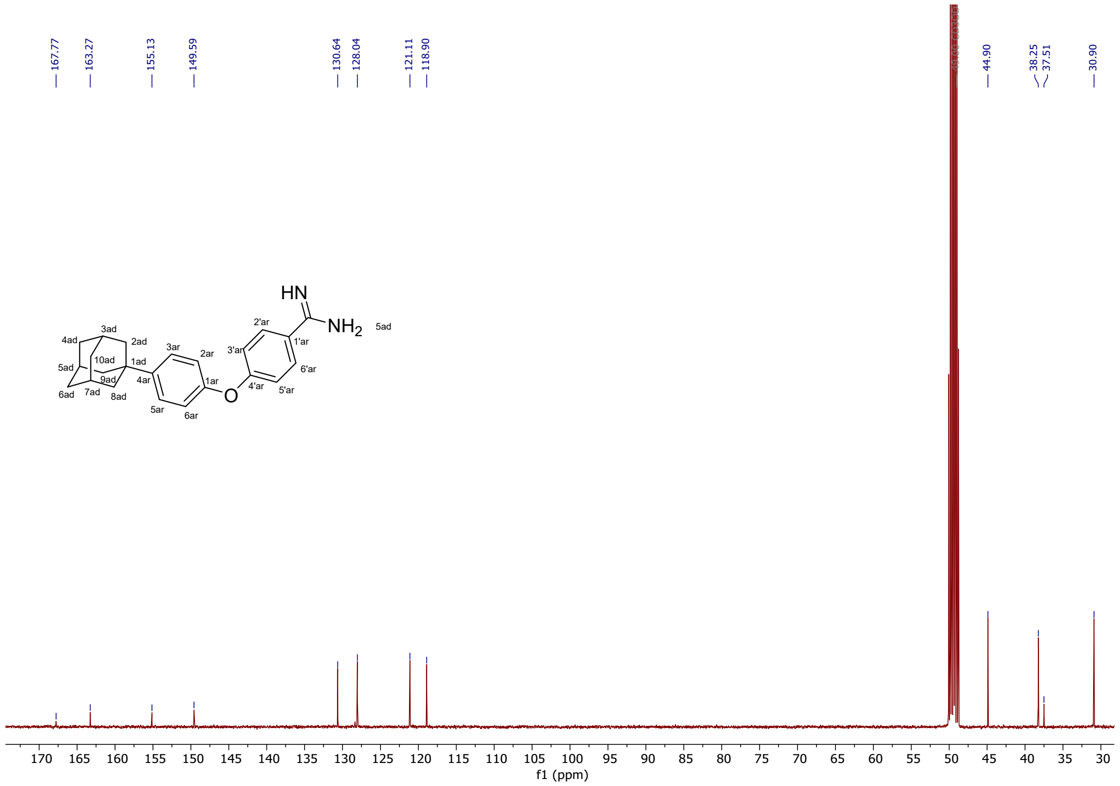

^{13}C NMR (101 MHz, CD_3OD) of 4-[4-(1-tricyclo[3.3.1.1^{3,7}]decyl)phenoxy]benzimidamide (3a).

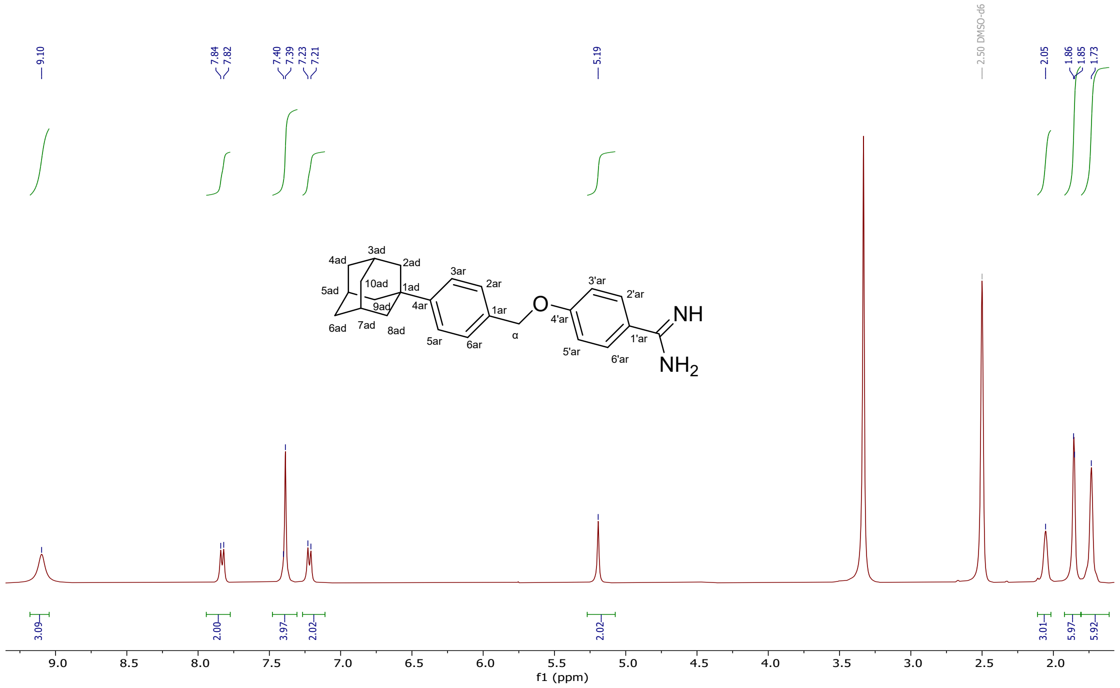

^{1}H NMR (400 MHz, DMSO-d_6) of 4-{[4-(1-tricyclo[3.3.1.1^{3,7}]decyl)benzyl]oxy}benzimidamide (3b).

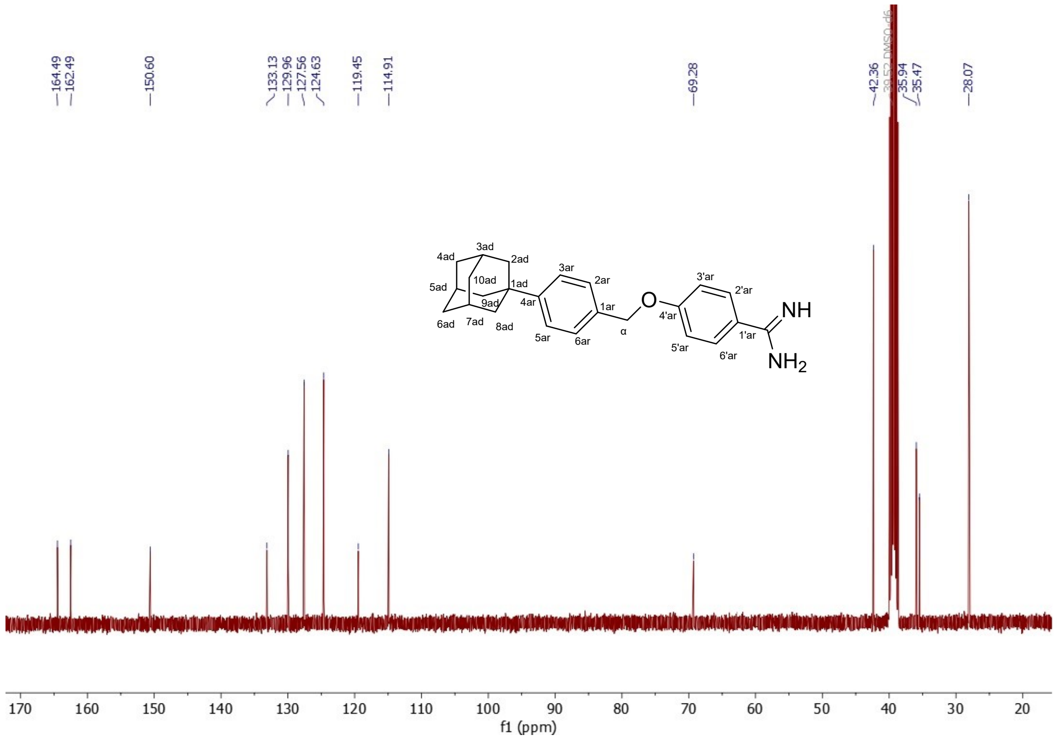

^{13}C NMR (101 MHz, DMSO-d_6) of 4-{[4-(1-tricyclo[3.3.1.1^{3,7}]decyl)benzyl]oxy}benzimidamide (3b).

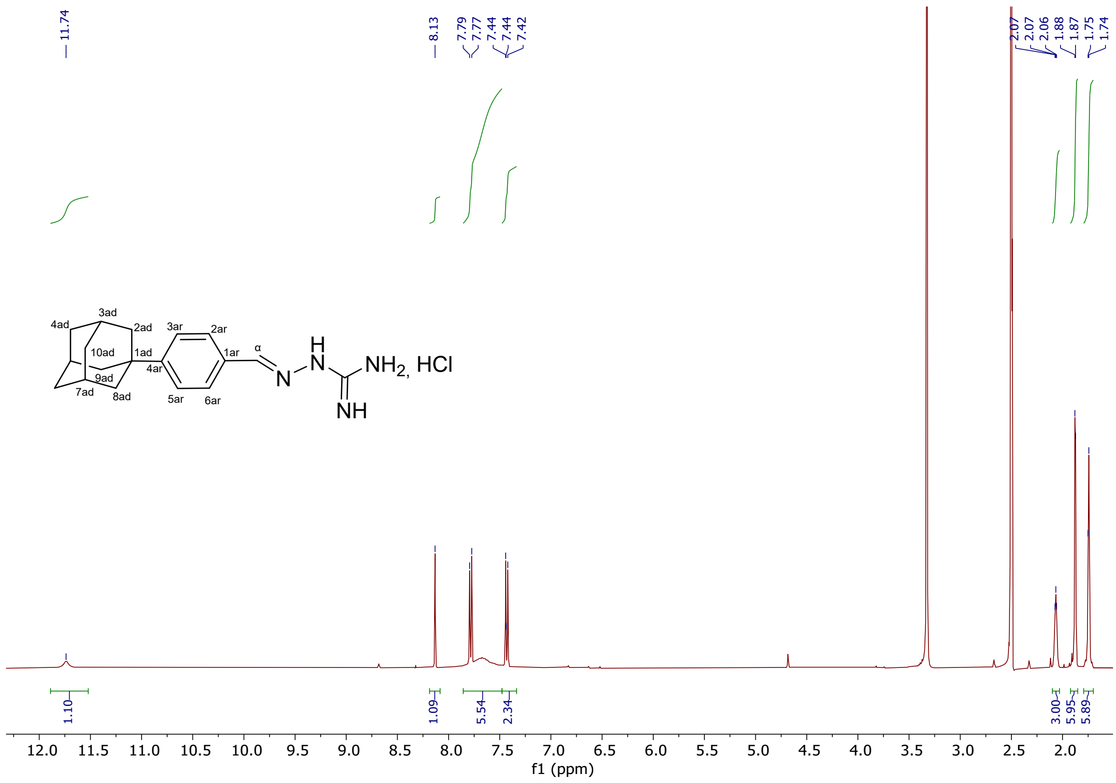

^{1}H NMR (400 MHz, DMSO-d_6) of 2-(*E*)-4-[(1-tricyclo[3.3.1.1^{3,7}]decyl)benzylidene]hydrazine-1-carboxyimidamide hydrochloride (4a).

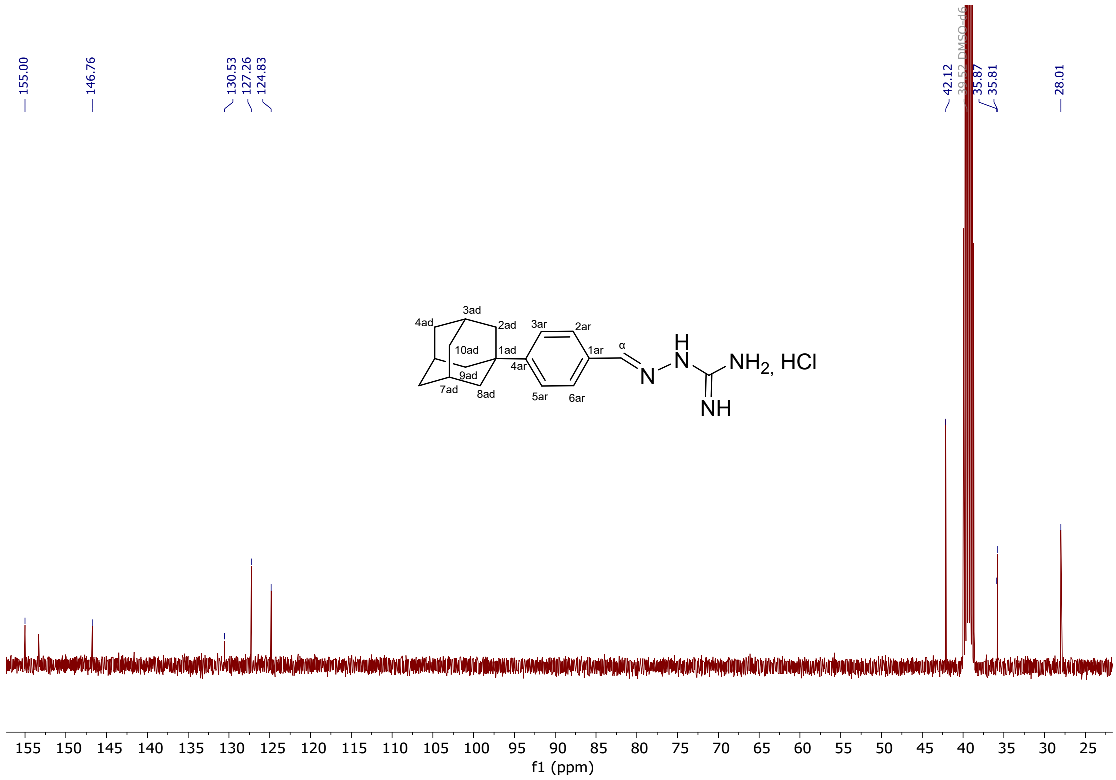

^{13}C NMR (101 MHz, DMSO-d_6) of 2-(*E*)-4-[(1-tricyclo[3.3.1.1^{3,7}]decyl)benzylidene]hydrazine-1-carboxyimidamide hydrochloride (4a).

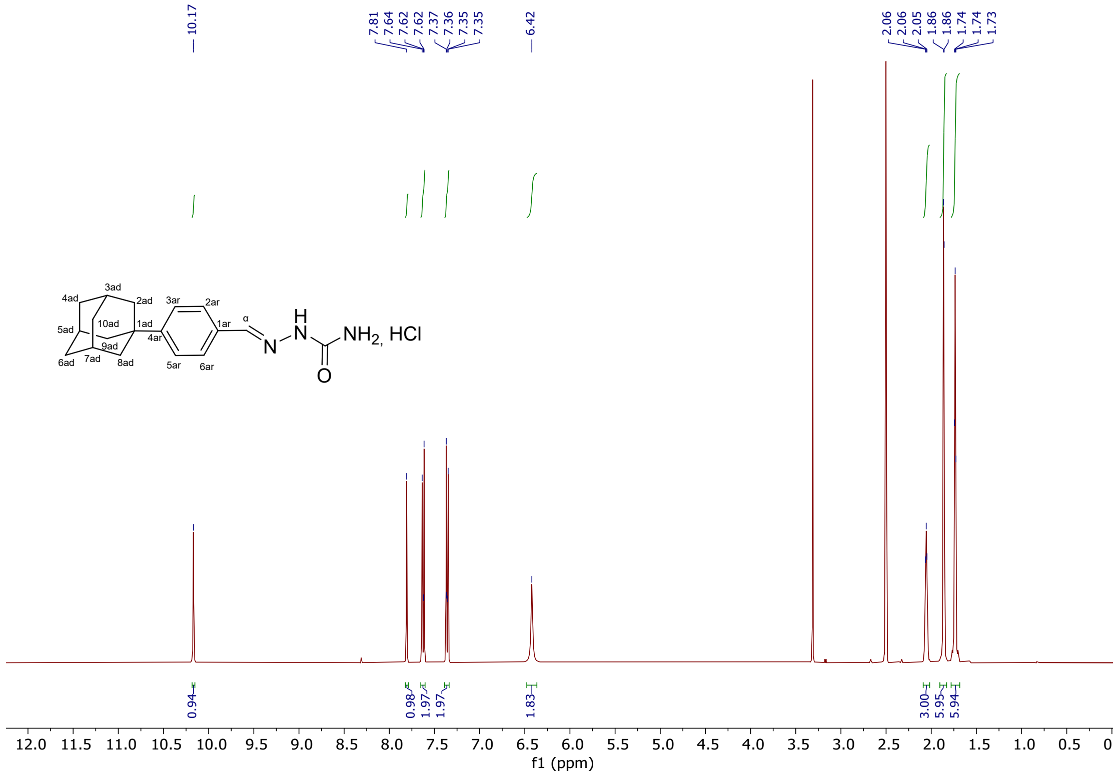

^{1}H NMR (400 MHz, DMSO-d_6) of 2-{(*E*)-4-(1-(tricyclo[3.3.1.1^{3,7}]decyl))benzylidene}hydrazine-1-carboxamide hydrochloride (4b).

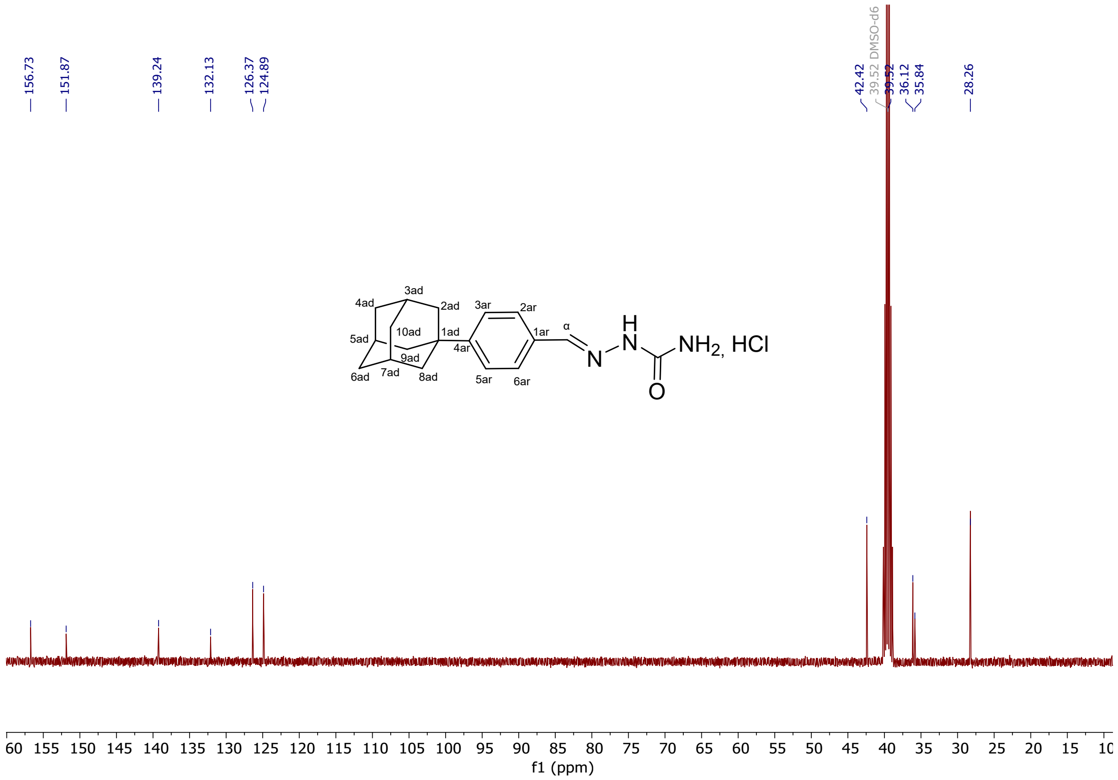

^{13}C NMR (101 MHz, DMSO-d_6) of 2-{(*E*)-4-(1-(tricyclo[3.3.1.1^{3,7}]decyl))benzylidene}hydrazine-1-carboxamide hydrochloride (4b).

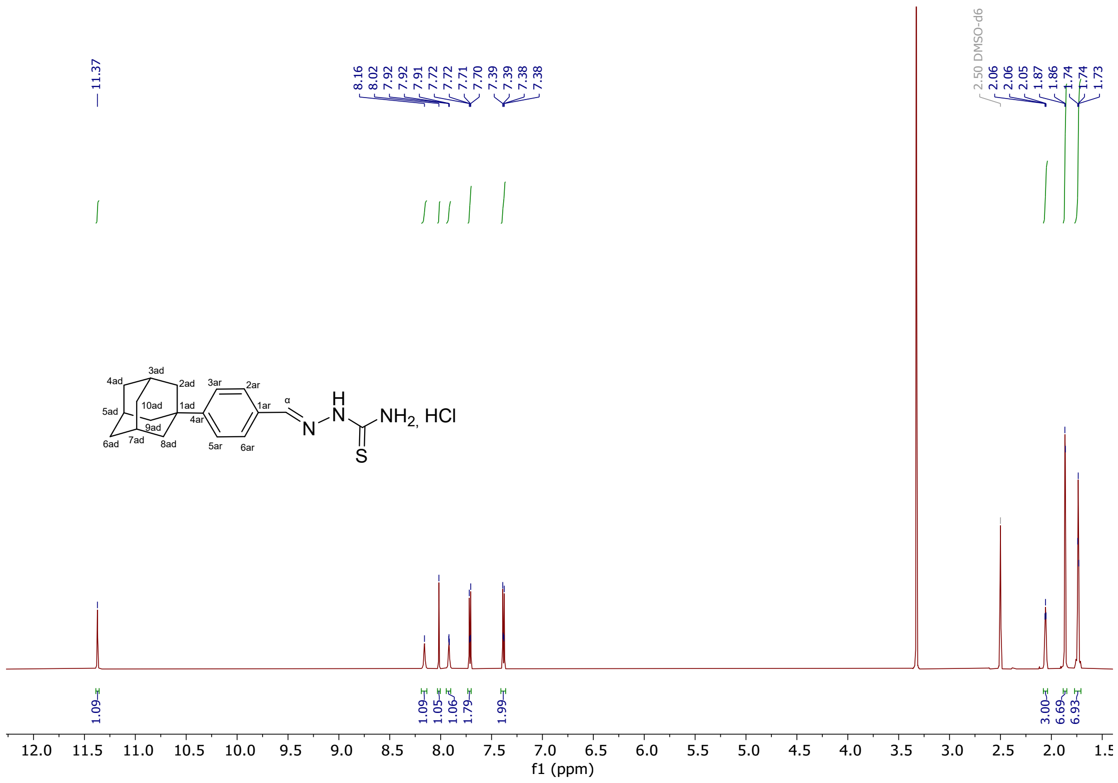

^{1}H NMR (600 MHz, DMSO-d_6) 2-(*E*)-4-[(1-tricyclo[3.3.1.1^{3,7}]decyl)benzylidene]hydrazine-1-carbothiamide hydrochloride (4c).

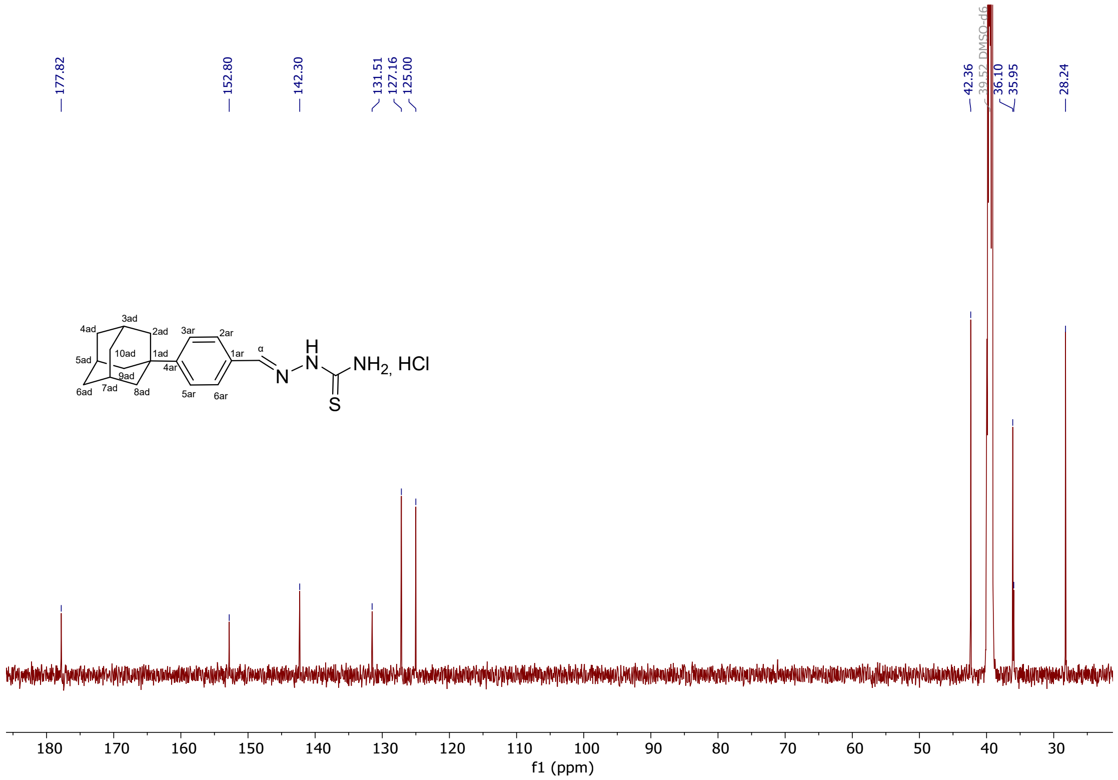

^{13}C NMR (151 MHz, DMSO-d_6) of 2-(*E*)-4-[(1-tricyclo[3.3.1.1^{3,7}]decyl)benzylidene]hydrazine-1-carbothiamide hydrochloride (4c).

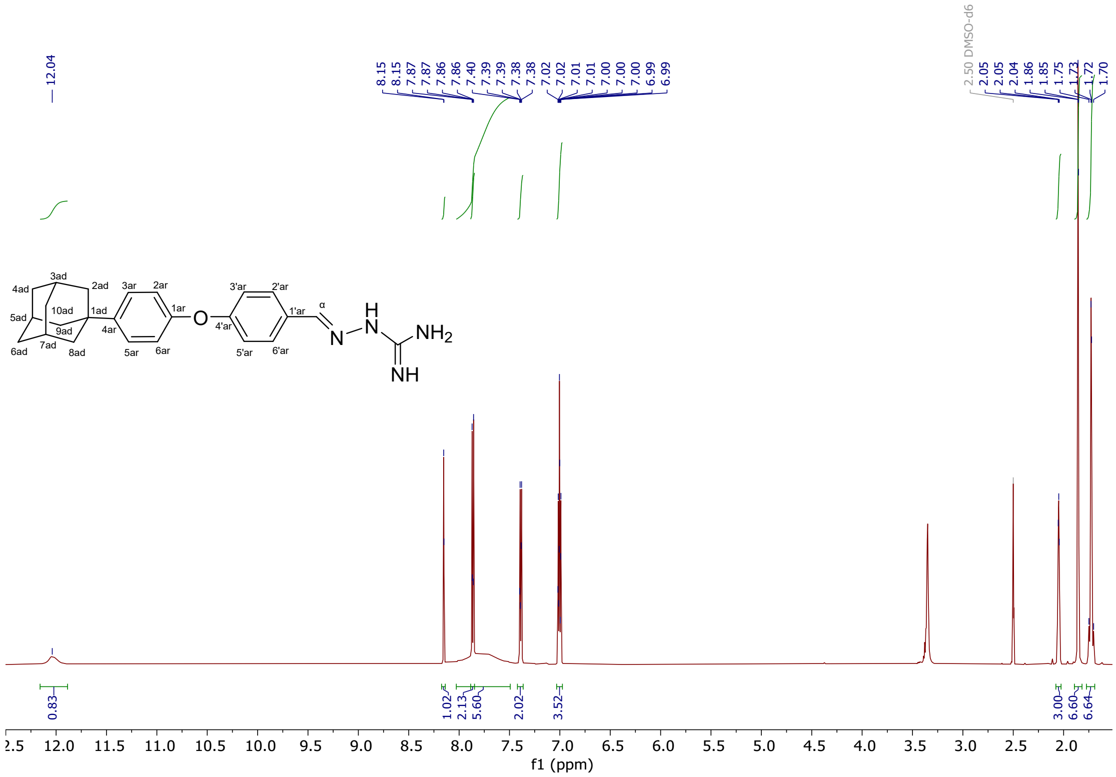

^{1}H NMR (600 MHz, DMSO-d_6) of 2-{(*E*)-4-[4-(1-tricyclo[3.3.1.1^{3,7}]decyl)phenoxy]benzylidene}hydrazine-1-carboxyimidamide hydrochloride (5a).

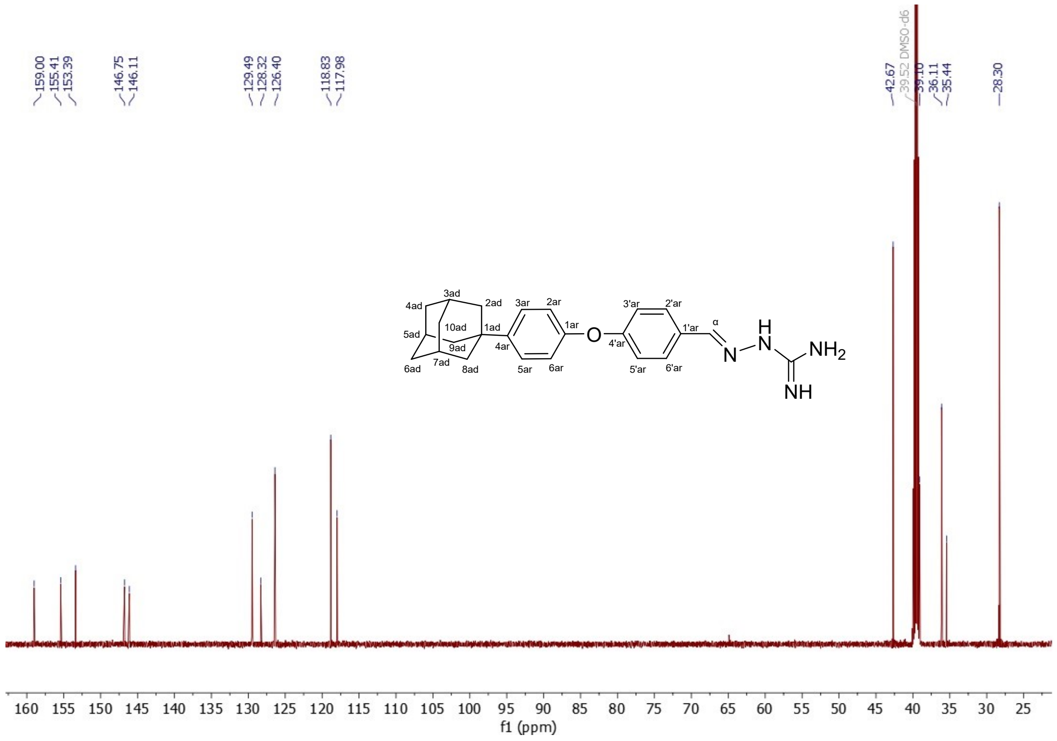

^{13}C NMR (151 MHz, DMSO-d_6) of 2-{(*E*)-4-[4-(1-tricyclo[3.3.1.1^{3,7}]decyl)phenoxy]benzylidene}hydrazine-1-carboxyimidamide hydrochloride (5a).

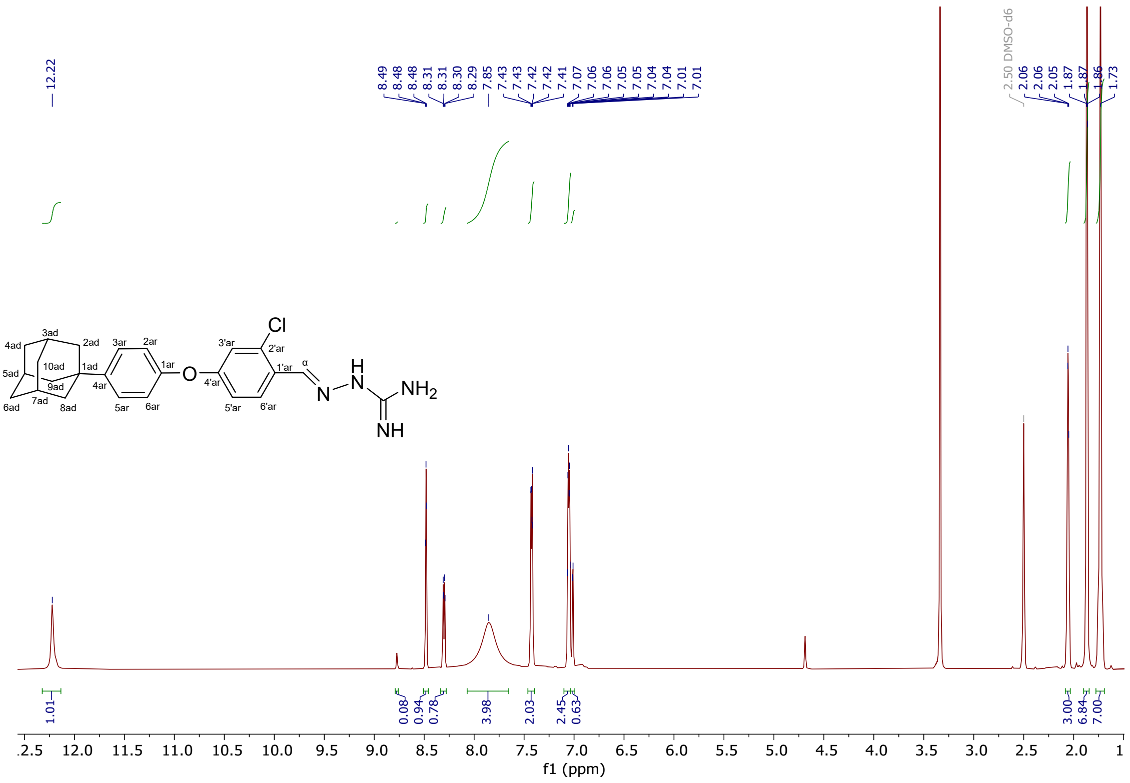

^{1}H NMR (600 MHz, DMSO-d_6) of 2-{(*E*)-4-[4-(1-tricyclo[3.3.1.1^{3,7}]decyl)phenoxy]-2-chlorobenzylidene}hydrazine-1-carboxyimidamide hydrochloride (5b).

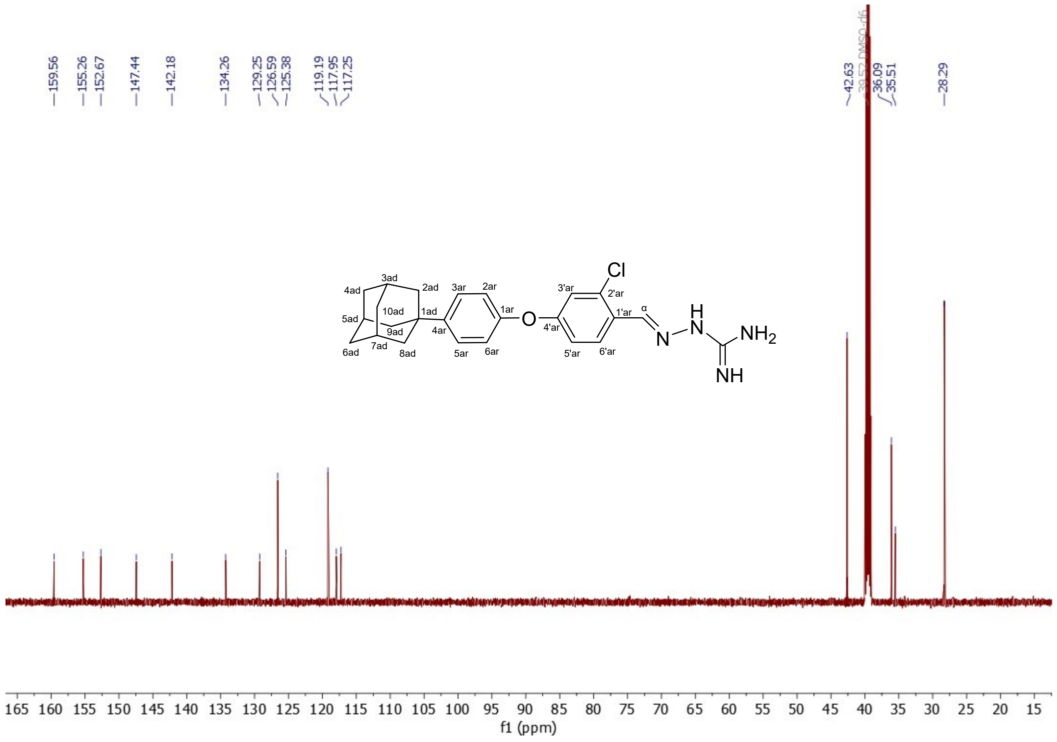

^{13}C NMR (151 MHz, DMSO-d_6) of 2-{(*E*)-4-[4-(1-tricyclo[3.3.1.1^{3,7}]decyl)phenoxy]-2-chlorobenzylidene}hydrazine-1-carboxyimidamide hydrochloride (5b).

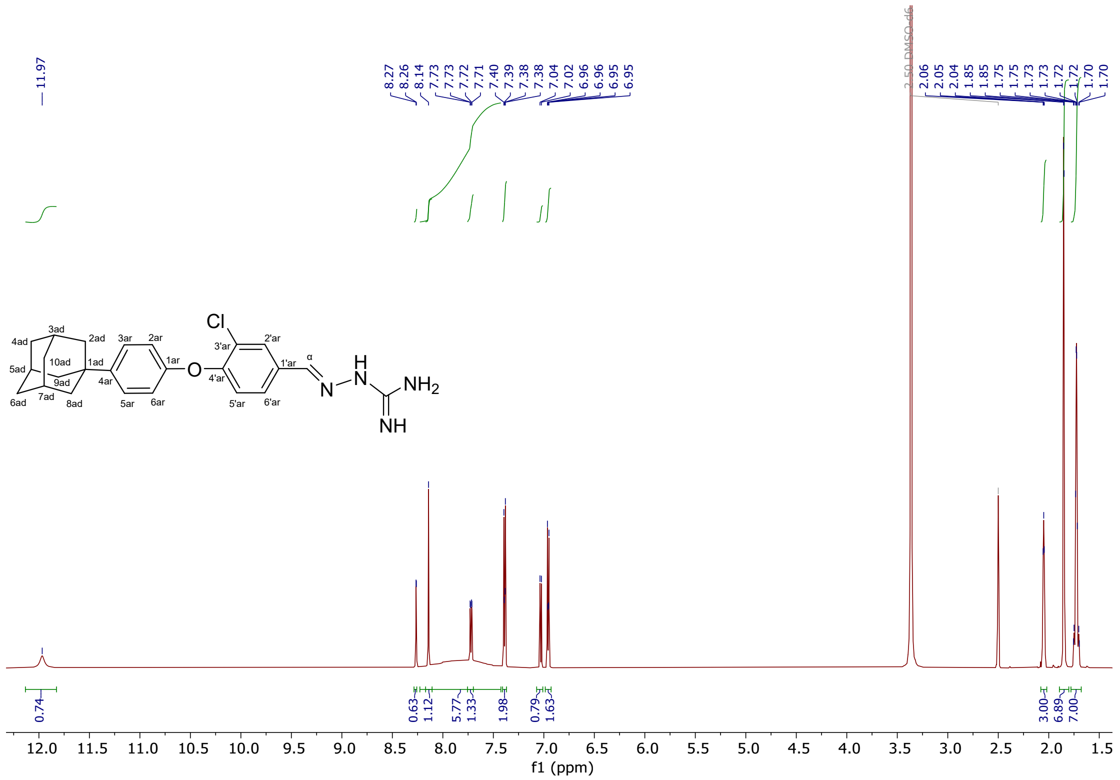

^{1}H NMR (600 MHz, DMSO-d_6) of 2-{(*E*)-4-[4-(1-tricyclo[3.3.1.1^{3,7}]decyl)phenoxy]-3-chlorobenzylidene}hydrazine-1-carboxyimidamide hydrochloride (5c).

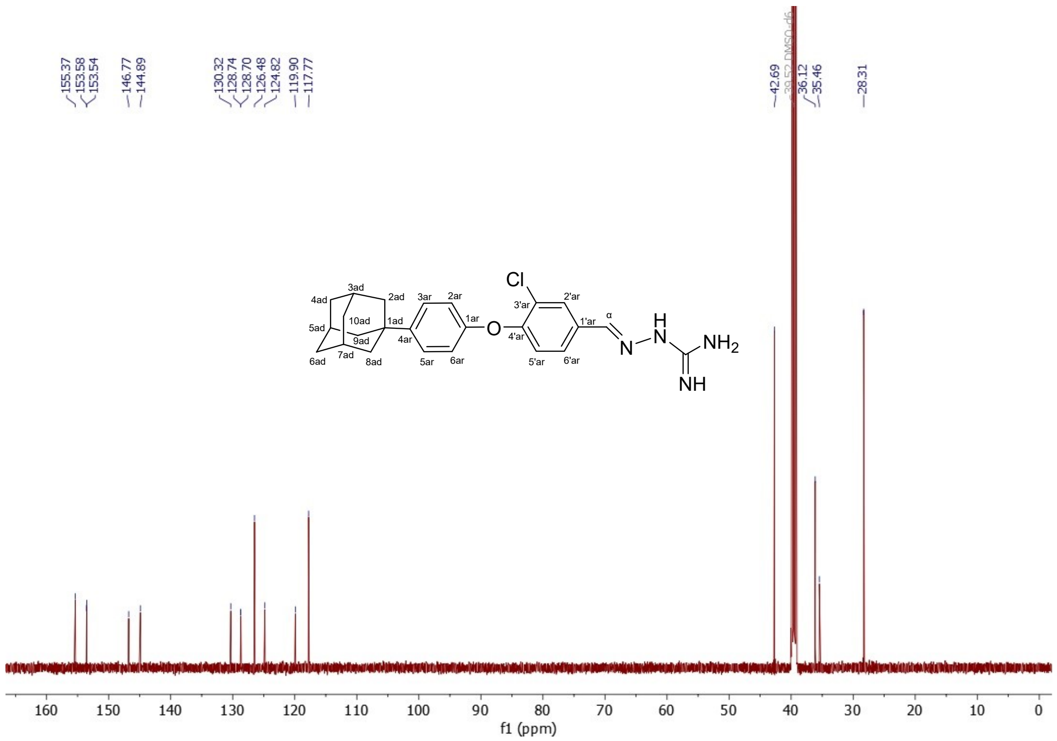

^{13}C NMR (151 MHz, DMSO-d_6) of 2-{(*E*)-4-[4-(1-tricyclo[3.3.1.1^{3,7}]decyl)phenoxy]-3-chlorobenzylidene}hydrazine-1-carboxyimidamide hydrochloride (5c).

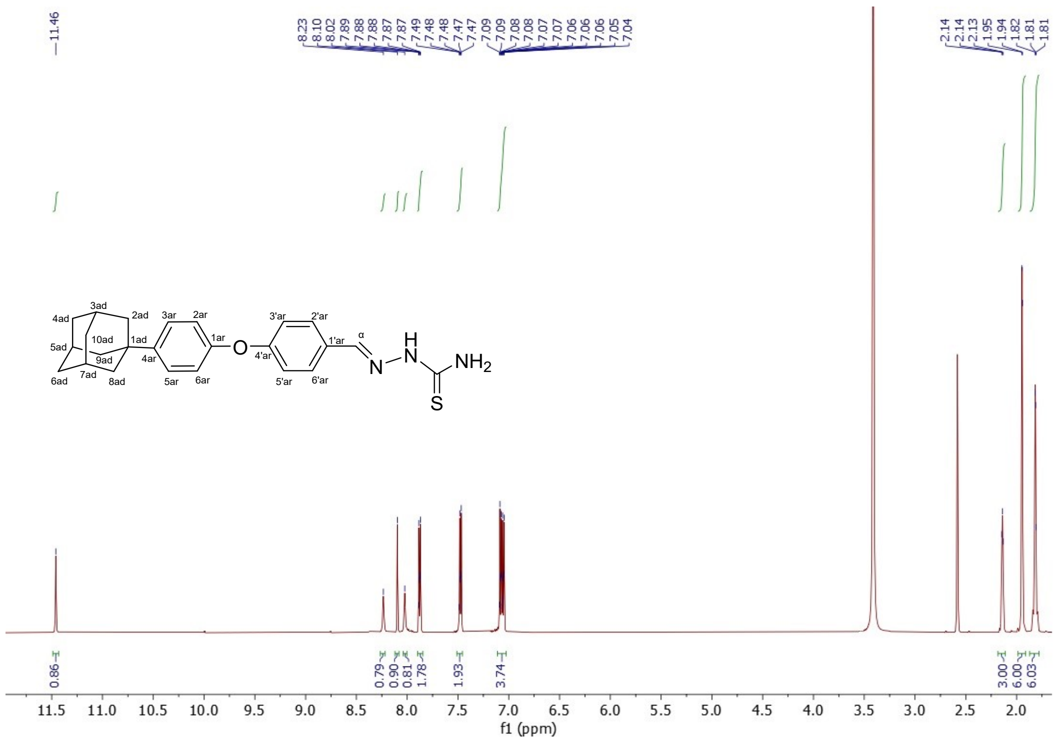

^{1}H NMR (600 MHz, DMSO-d_6) of 2-{(*E*)-4-[4-(1-tricyclo[3.3.1.1^{3,7}]decyl)phenoxy)benzylidene}hydrazine-1-carbothioamide hydrochloride (6a).

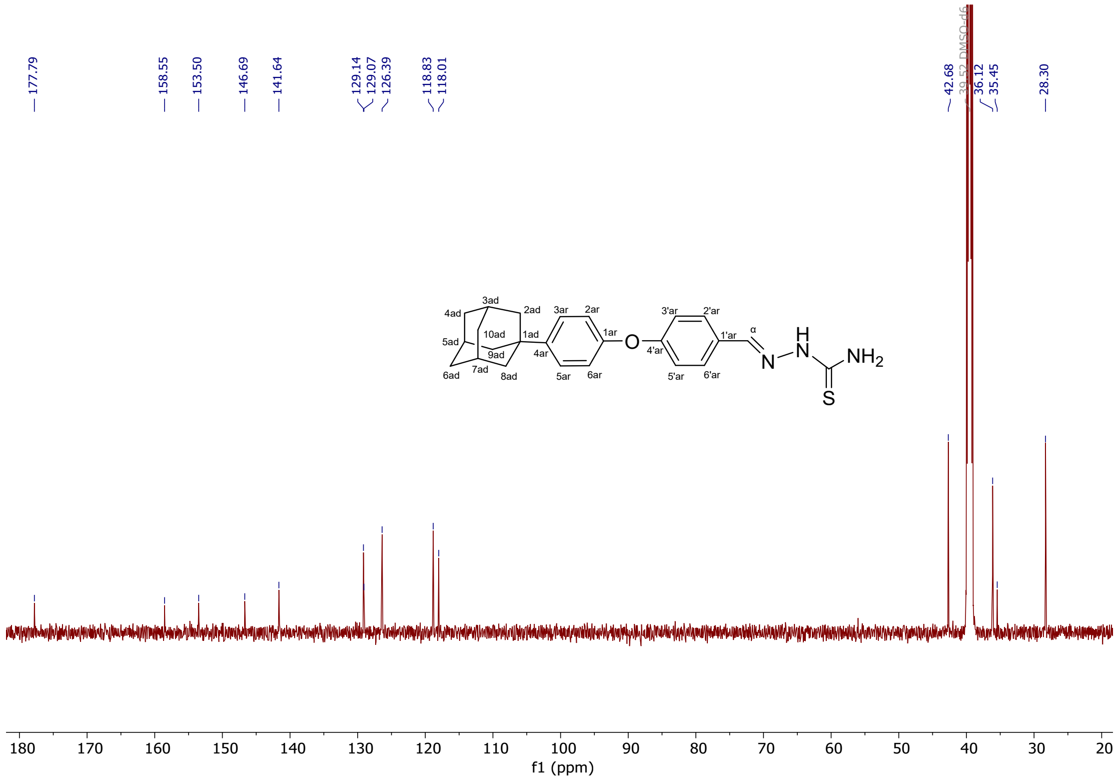

^{13}C NMR (151 MHz, DMSO-d_6) of 2-{(*E*)-4-[4-(1-tricyclo[3.3.1.1^{3,7}]decyl)phenoxy)benzylidene}hydrazine-1-carbothioamide hydrochloride (6a).

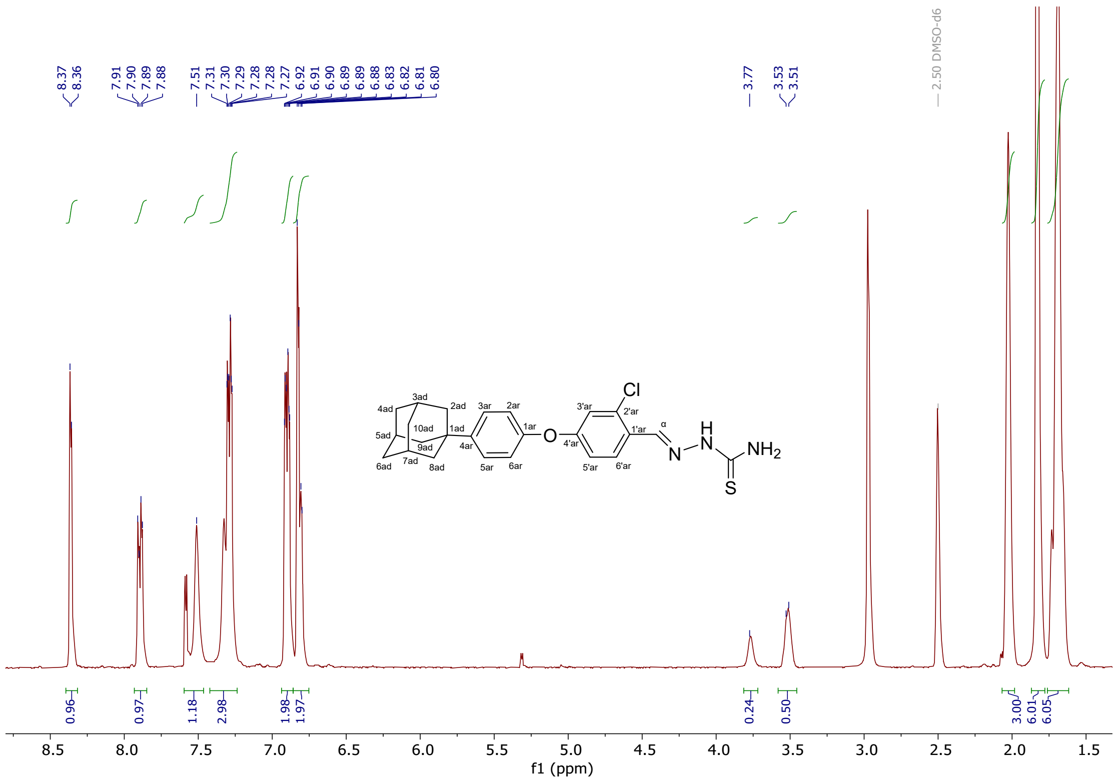

^{1}H NMR (400 MHz, DMSO-d_6) of 2-{(*E*)-4-(4-(1-tricyclo[3.3.1.1^{3,7}]decyl)phenoxy)-2-chlorobenzylidene}hydrazine-1-carbothioamide hydrochloride (6b).

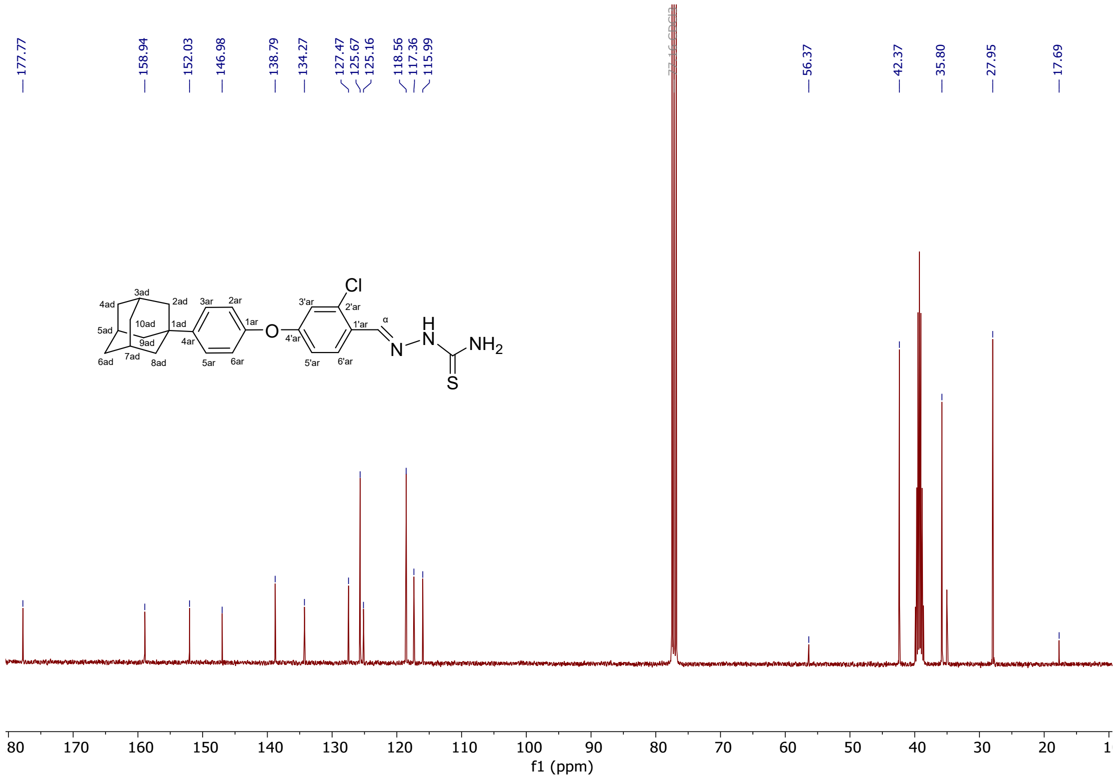

^{13}C NMR (101 MHz, DMSO-d_6) of 2-{(*E*)-4-(4-(1-tricyclo[3.3.1.1^{3,7}]decyl)phenoxy)-2-chlorobenzylidene}hydrazine-1-carbothioamide hydrochloride (6b).

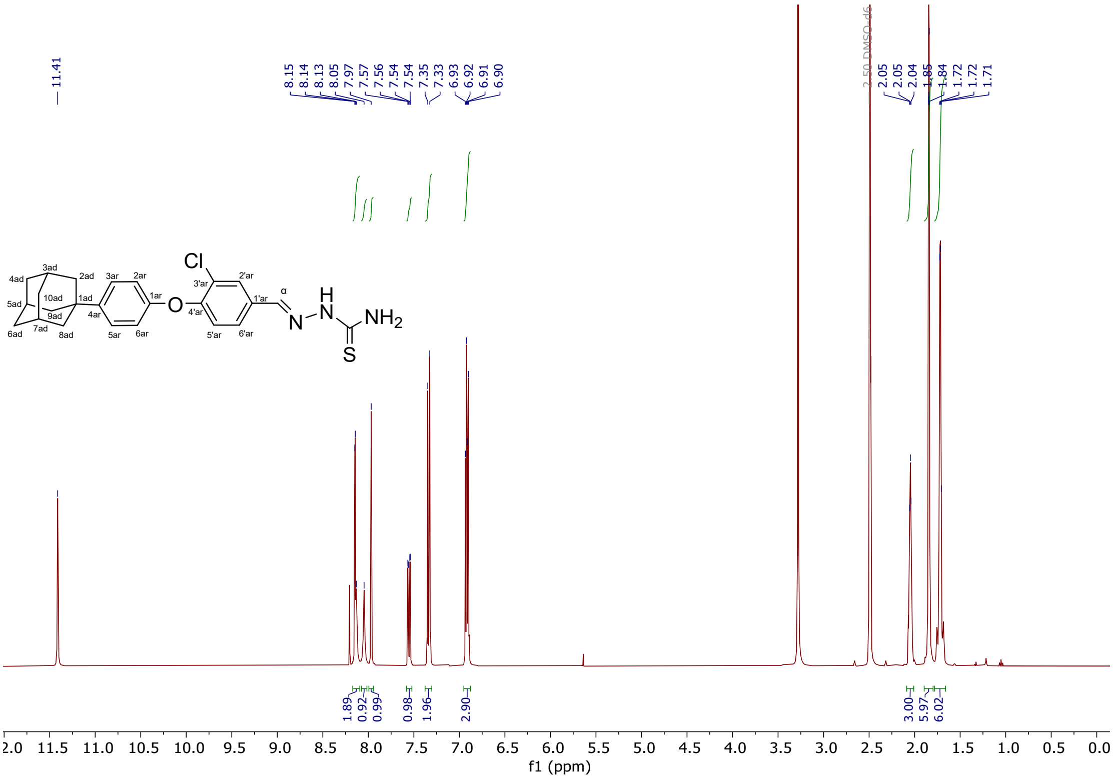

^{1}H NMR (400 MHz, $CDCl_3$+DMSO-d_6) of 2-{(*E*)-4-[4-(1-tricyclo[3.3.1.1^{3,7}]decyl)phenoxy]-3-chlorobenzylidene}hydrazine-1-carbothioamide hydrochloride (6c).

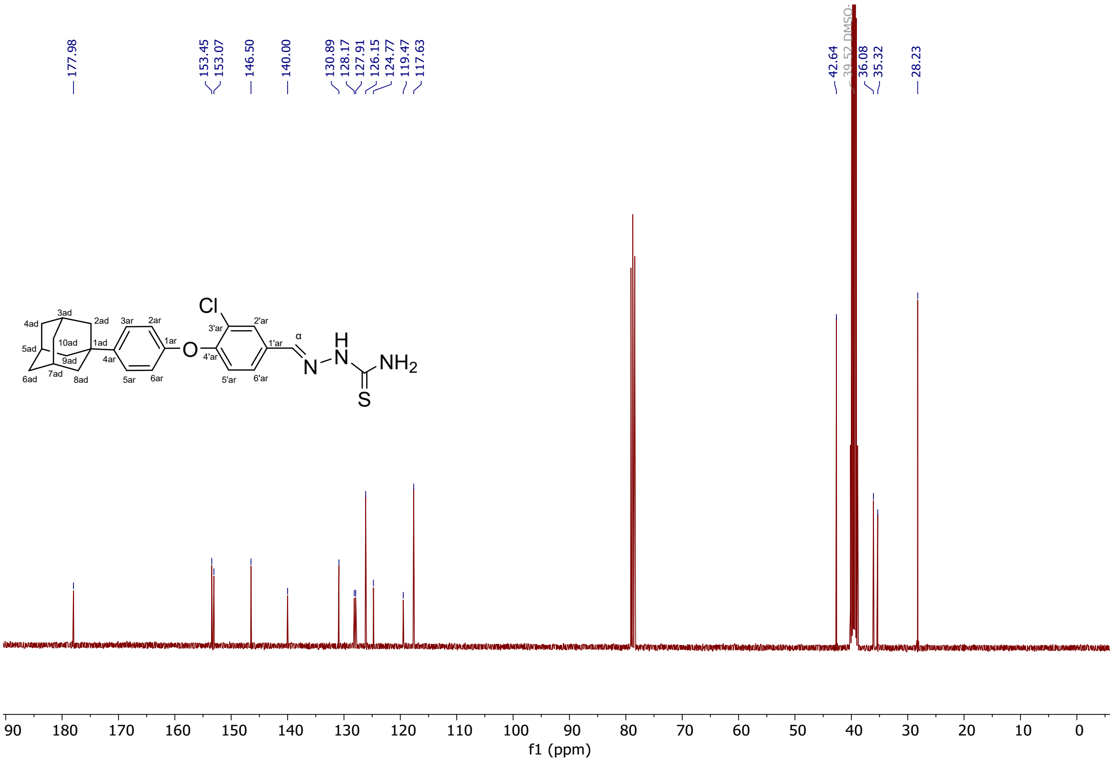

^{13}C NMR (101 MHz, $CDCl_3$+DMSO-d_6) of 2-{(*E*)-4-[4-(1-tricyclo[3.3.1.1^{3,7}]decyl)phenoxy]-3-chlorobenzylidene}hydrazine-1-carbothioamide hydrochloride (6c).

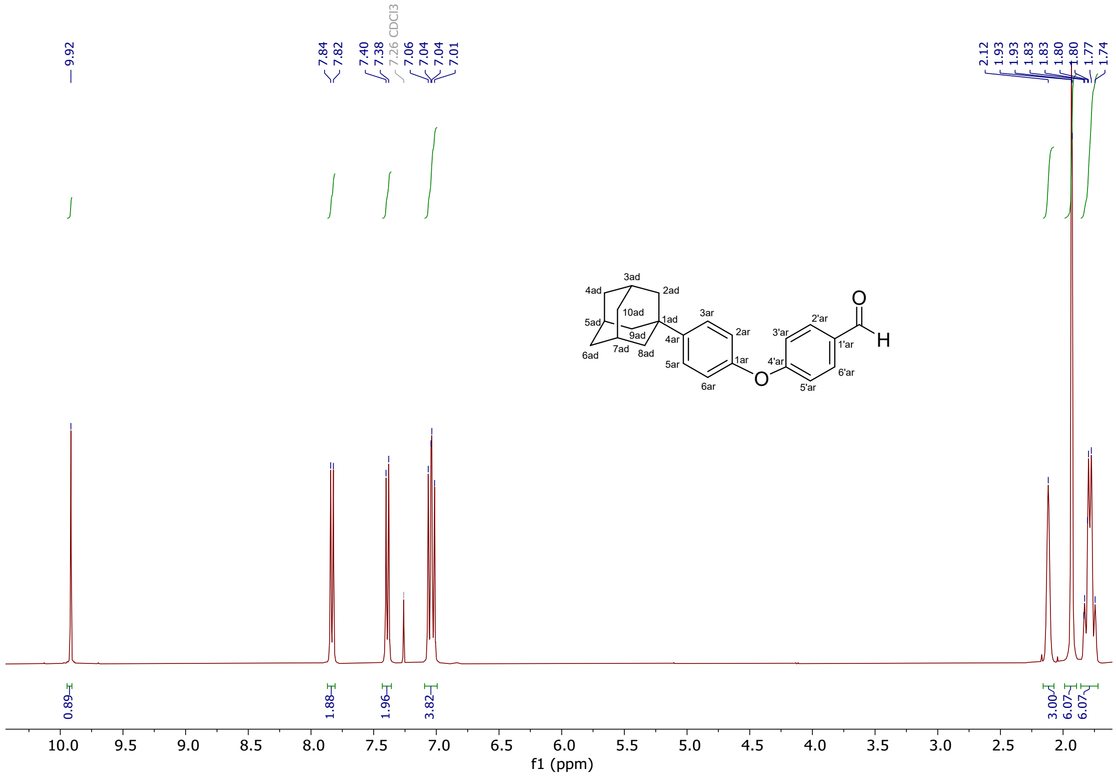

^{1}H NMR (400 MHz, $CDCl_3$) of 4-[4-(1-tricyclo[3.3.1.1^{3,7}]decyl)phenoxy]benzaldehyde (21).

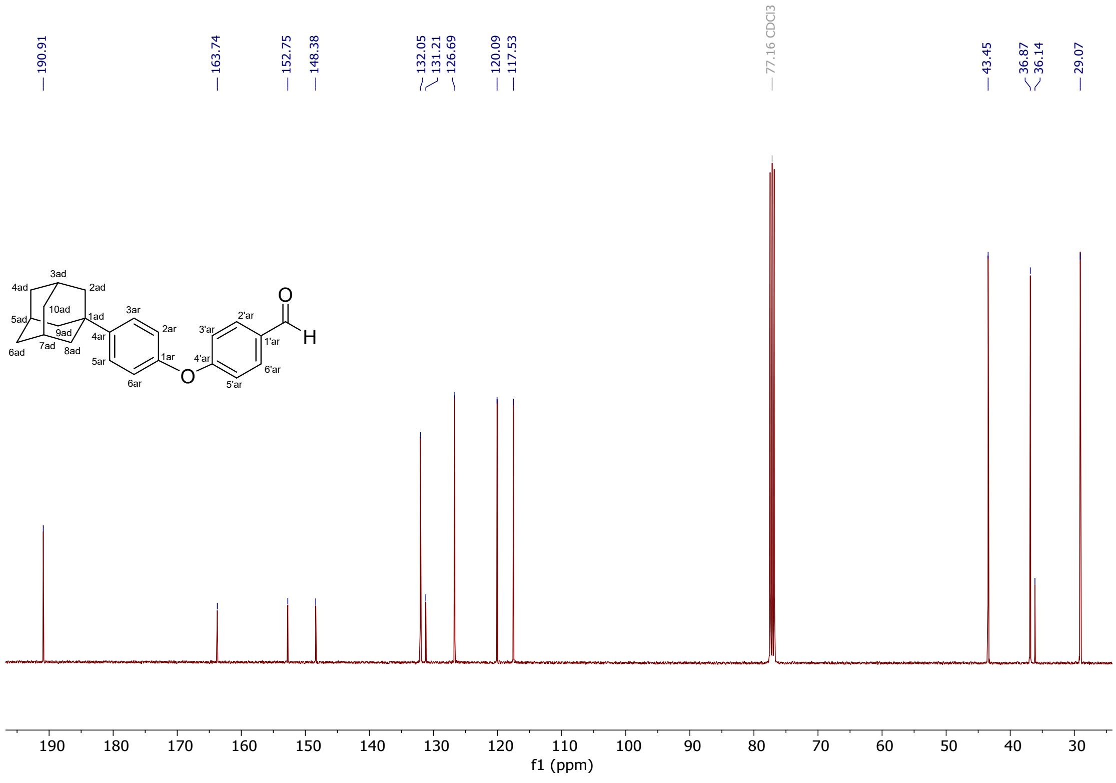

^{13}C NMR (101 MHz, $CDCl_3$) of 4-[4-(1-tricyclo[3.3.1.1^{3,7}]decyl)phenoxy]benzaldehyde (21).

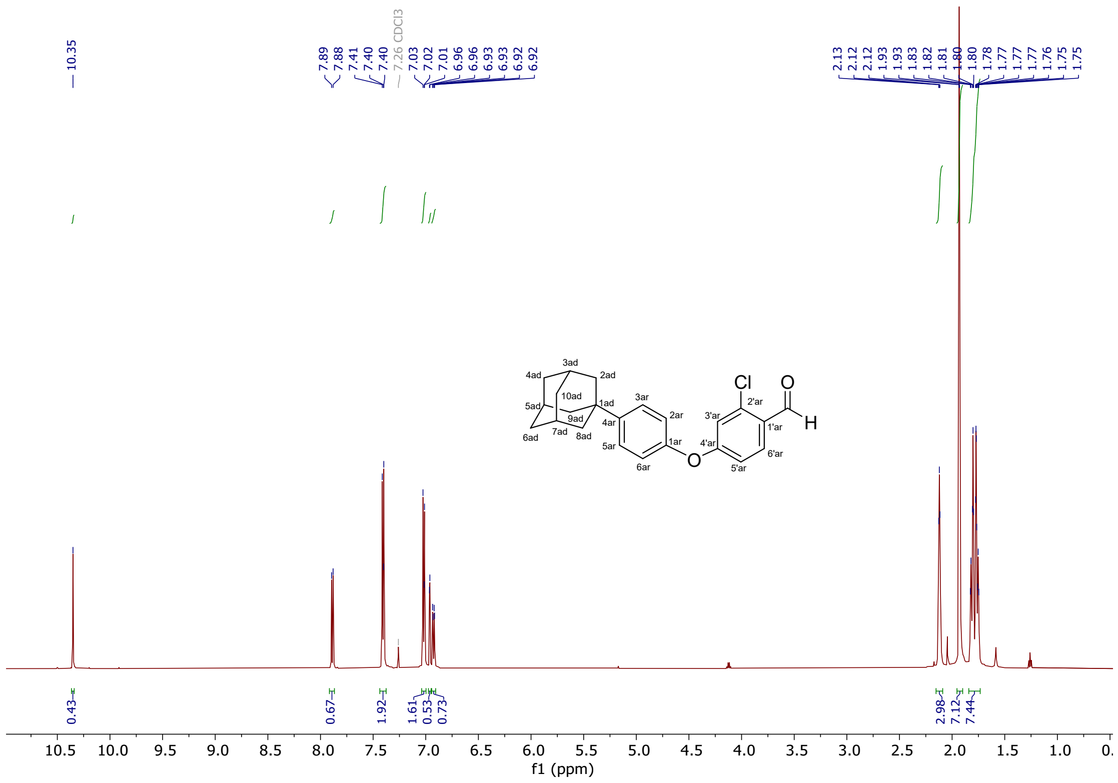

^{1}H NMR (600 MHz, DMSO-d_6) of 4-[4-(1-tricyclo[3.3.1.1^{3,7}]decyl)phenoxy]-2-chlorobenzaldehyde (22).

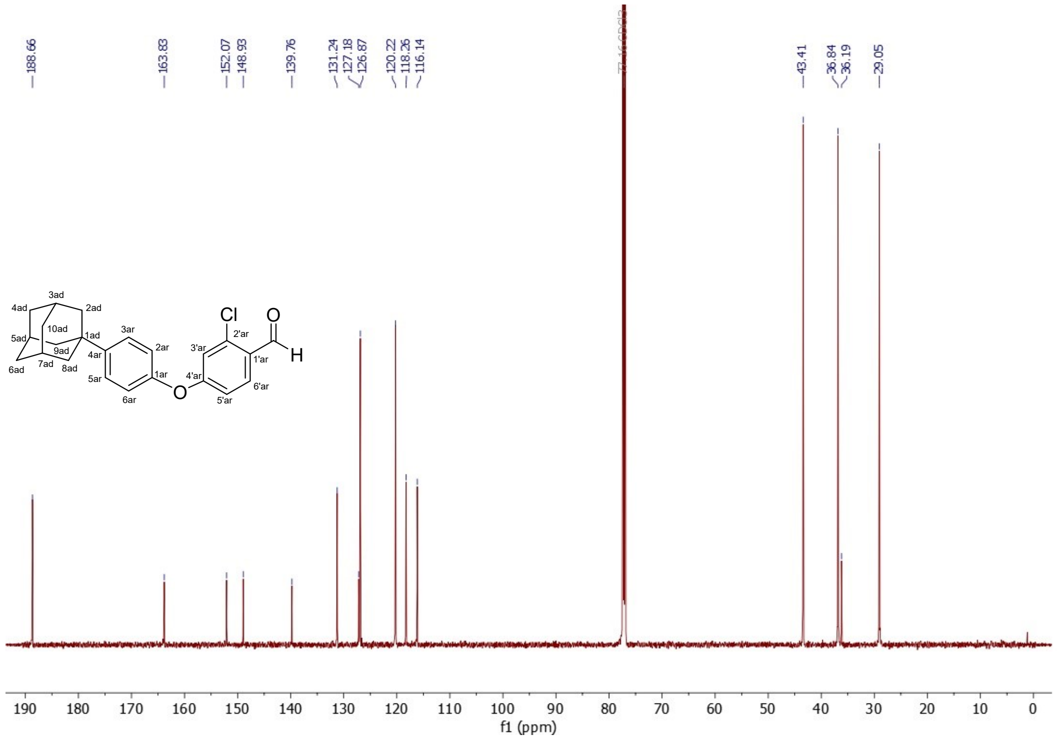

^{13}C NMR (151 MHz, DMSO-d_6) of 4-[4-(1-tricyclo[3.3.1.1^{3,7}]decyl)phenoxy]-2-chlorobenzaldehyde (22).

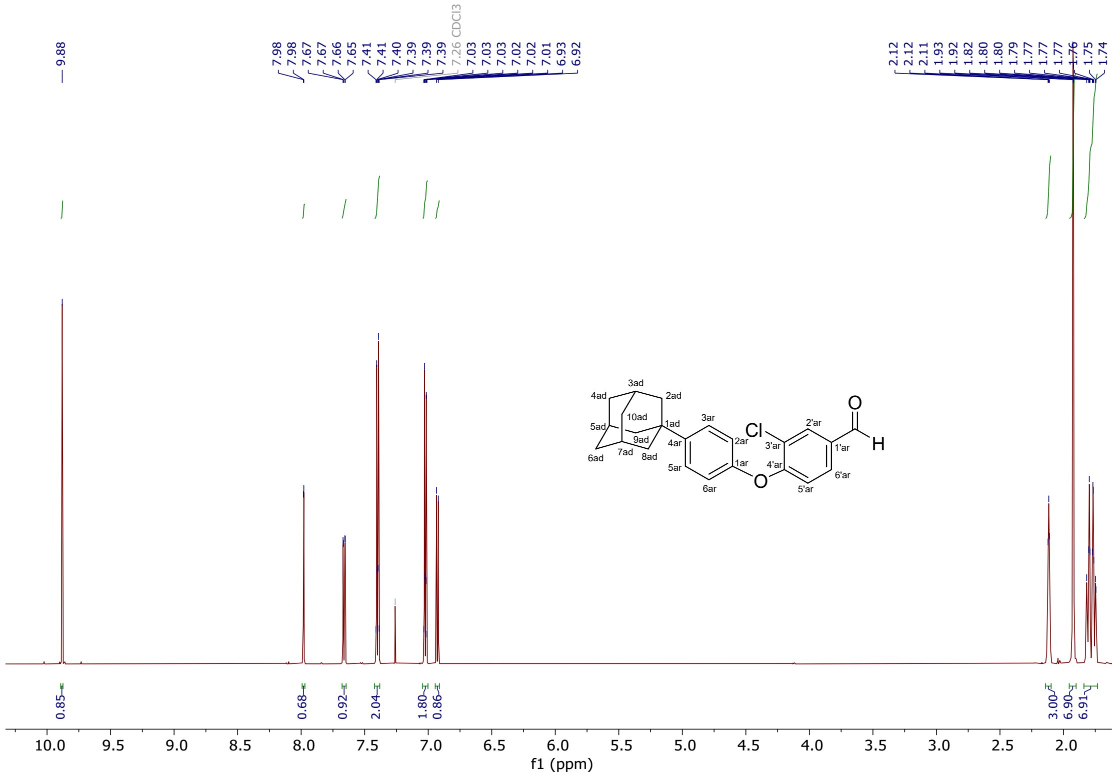

^{1}H NMR (600 MHz, $CDCl_3$) of 4-[4-(1-tricyclo[3.3.1.1^{3,7}]decyl)phenoxy]-3-chlorobenzaldehyde (23).

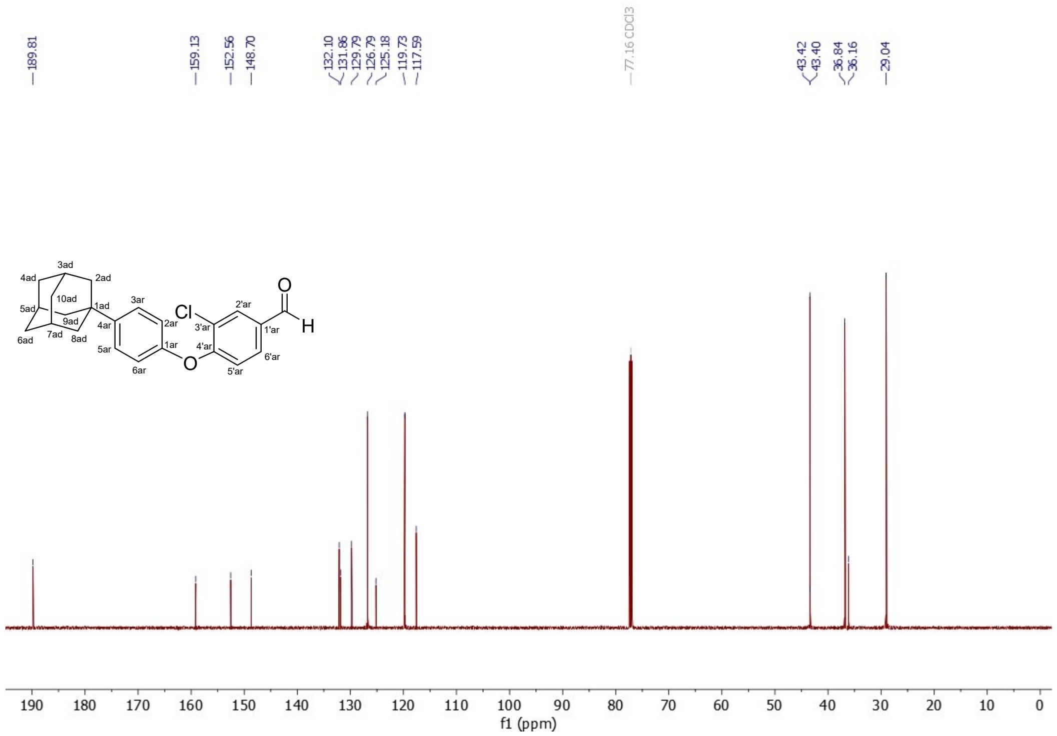

^{13}C NMR (151 MHz, $CDCl_3$) of 4-[4-(1-tricyclo[3.3.1.1^{3,7}]decyl)phenoxy]-3-chlorobenzaldehyde (23).
